# Oxalate production via oxidation of ascorbate rather than reduction of carbon dioxide

**DOI:** 10.1038/s41467-021-21817-w

**Published:** 2021-03-25

**Authors:** Fatemeh Khamespanah, Maximilian Marx, David B. Crochet, Uttam R. Pokharel, Frank R. Fronczek, Andrew W. Maverick, Matthias Beller

**Affiliations:** 1 Department of Chemistry, Louisiana State University, Baton Rouge, LA USA; 2 Leibniz Institute for Catalysis at the University of Rostock, Rostock, Germany; 3 Present Address: Department of Chemistry and Physical Sciences, Nicholls State University, Thibodaux, LA USA

**Keywords:** Inorganic chemistry, Self-assembly, Energy science and technology

**Arising from** Pokharel et al. *Nature Communications* 10.1038/ncomms6883 (2014)

In the previous publication, some of us reported the conversion of a copper(I) complex to a copper(II) oxalate complex, and claimed that this conversion involved a reduction of CO_2_ to oxalate (C_2_O_4_^2−^). Herein, we show that the oxalate is produced not by reduction of CO_2_, but by reaction of ascorbate with oxygen. We also present new results that explain in a more comprehensive way the behaviour of these copper compounds under O_2_ and CO_2_.

Selective reduction of carbon dioxide to C_≥2_ compounds using homogeneous metal complexes is a challenging transformation. Only a limited number of examples have been reported over the past decades^1–12^. In contrast, there has been a vast increase in reported catalysts for selective CO_2_ reduction to C_1_ compounds^13–15^. Among the examples reported for the reductive coupling of CO_2_ to oxalate is a dinuclear Cu complex introduced by some of us in 2014 (ref. ^16^). The in situ generated Cu(I) complex [Cu_2_(*m*-xpt)_2_](PF_6_)_2_ (**3**) formed by reduction of the Cu(II) precursor (**1**) with sodium ascorbate generated an oxalate-bridged dinuclear complex (**4**), proposed to occur via reductive coupling of atmospheric CO_2_ (Fig. 1). Release of the oxalate by addition of mineral acids was described, potentially enabling stepwise conversion of CO_2_ into oxalic acid using sodium ascorbate as a comparatively mild reductant.

**Fig. 1:**
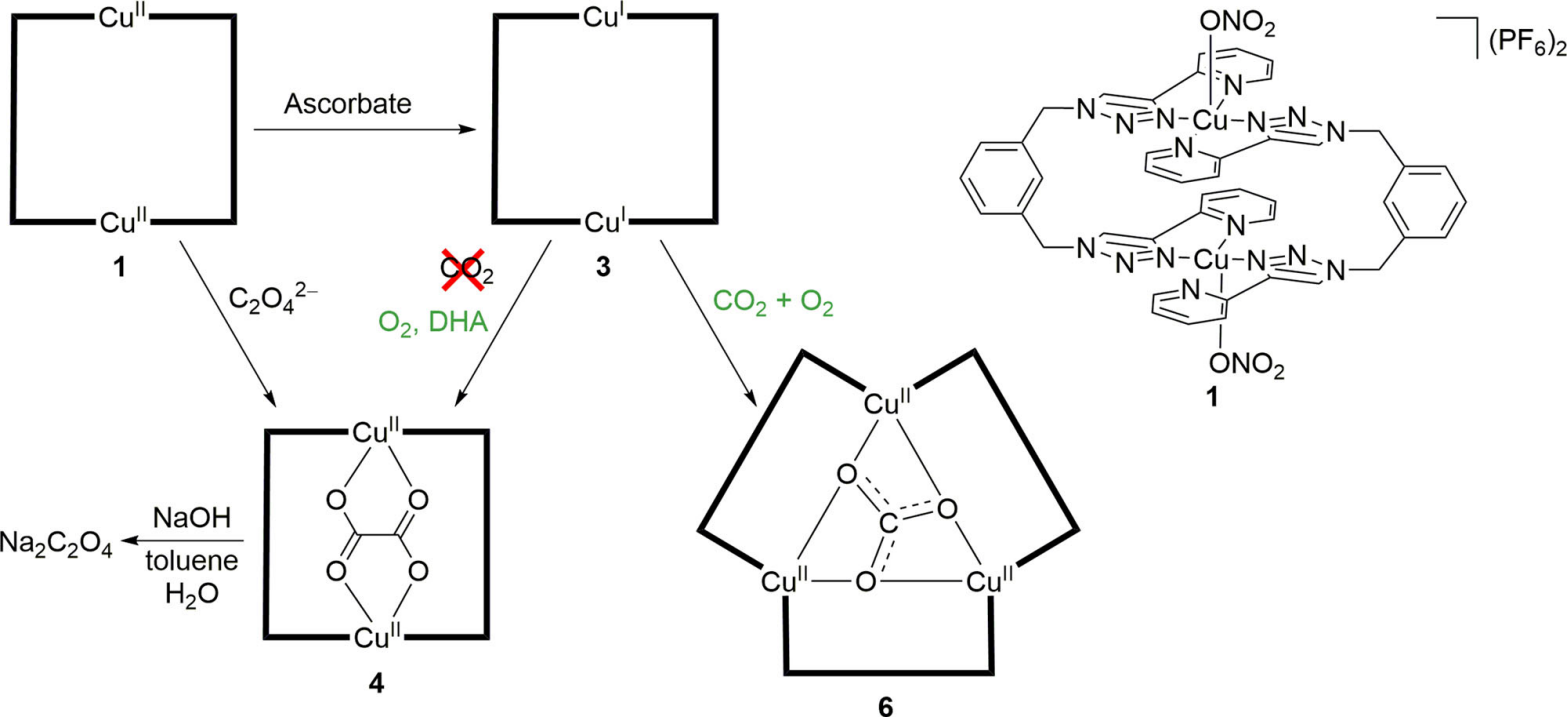
Reactions of Cu(II) complex 1 and the Cu(I) complex 3 obtained by reduction of 1 with ascorbate. For the formation of oxalate complex **4** from **3**, CO_2_ was previously reported to be required. We show here that the reaction requires ascorbate or dehydroascorbic acid (DHA), and oxygen. If ascorbate and DHA are absent, oxidation of **3** in air produces **6**. All reactions were conducted in DMF, except for the removal of oxalate from **4** (Note: **1**, **3**, and **4** represent the same compounds as in ref. ^16^).

Interestingly, oxidation of ascorbic acid by transition metal compounds, especially those of copper, has been well-known for more than a century^17,18^. Since then, the reaction mechanisms for such oxidations have been intensely studied^18–22^. More specifically, oxidative degradation of ascorbic acid by (a) inorganic oxidants (sodium periodate^23^, sodium hypoiodite^24^); (b) oxygen^25,26^; and (c) O_2_ in the presence of Gd^27,28^, Co^27^, Pd^29^, Pt^29^, Cd^30^, Fe^31^, or Cu^32^ compounds is reported to yield oxalate as a degradation product (see Supplementary Fig. 21 for a typical reaction sequence).

We now report that the true origin of the oxalate in the communication published in 2014 is not CO_2_, as it was described, but oxidative degradation of sodium ascorbate.

A first hint towards the oxidative degradation pathway as the origin of oxalate was obtained when treatment of the in situ generated Cu(I) complex [Cu_2_(*m*-xpt)_2_](PF_6_)_2_ (**3**), formed via reaction of the Cu(II) precursor **1** with sodium ascorbate in DMF, with CO_2_ over 6 days did not result in the previously described colour change from yellow to green (Supplementary Fig. 1) and no Cu(II) species was detected by UV/Vis spectroscopy (Supplementary Fig. 2). However, after introduction of air, oxidation of the Cu(I) complex **3** was observed and followed by UV/Vis spectroscopy over 189 h, resembling the UV/Vis spectra reported in the previous publication.

The product obtained from this reaction after slow evaporation of the solvent was identical to the reported oxalate complex **4**, as evident from the IR spectrum (Supplementary Fig. 3).

Since the reaction seemed to require air for the formation of oxalate, we suggested that oxidation of the ascorbate might be the true origin of the oxalate. Therefore, the previously published results might have eventuated from oxygen contamination of the reaction mixtures utilised for the labelling studies and the UV/Vis spectroscopic study.

To test this hypothesis, we prepared the Cu(I) complex **3** in situ using sodium ascorbate, and exposed it to oxygen in the absence of air and CO_2_. Indeed, oxidation of the Cu(I) complex **3** in the presence of O_2_ occurred within a few minutes, as evidenced by a characteristic colour change from yellow to green (Supplementary Fig. 5) and after 5 days of reaction time, a yellow to green solid was obtained after removal of the solvent. As suspected, the solid product was identical to that obtained via the reaction of the in situ generated Cu(I) complex **3** with air, as evident from X-ray analysis and FTIR spectroscopy (see spectra in Fig. 2).

**Fig. 2:**
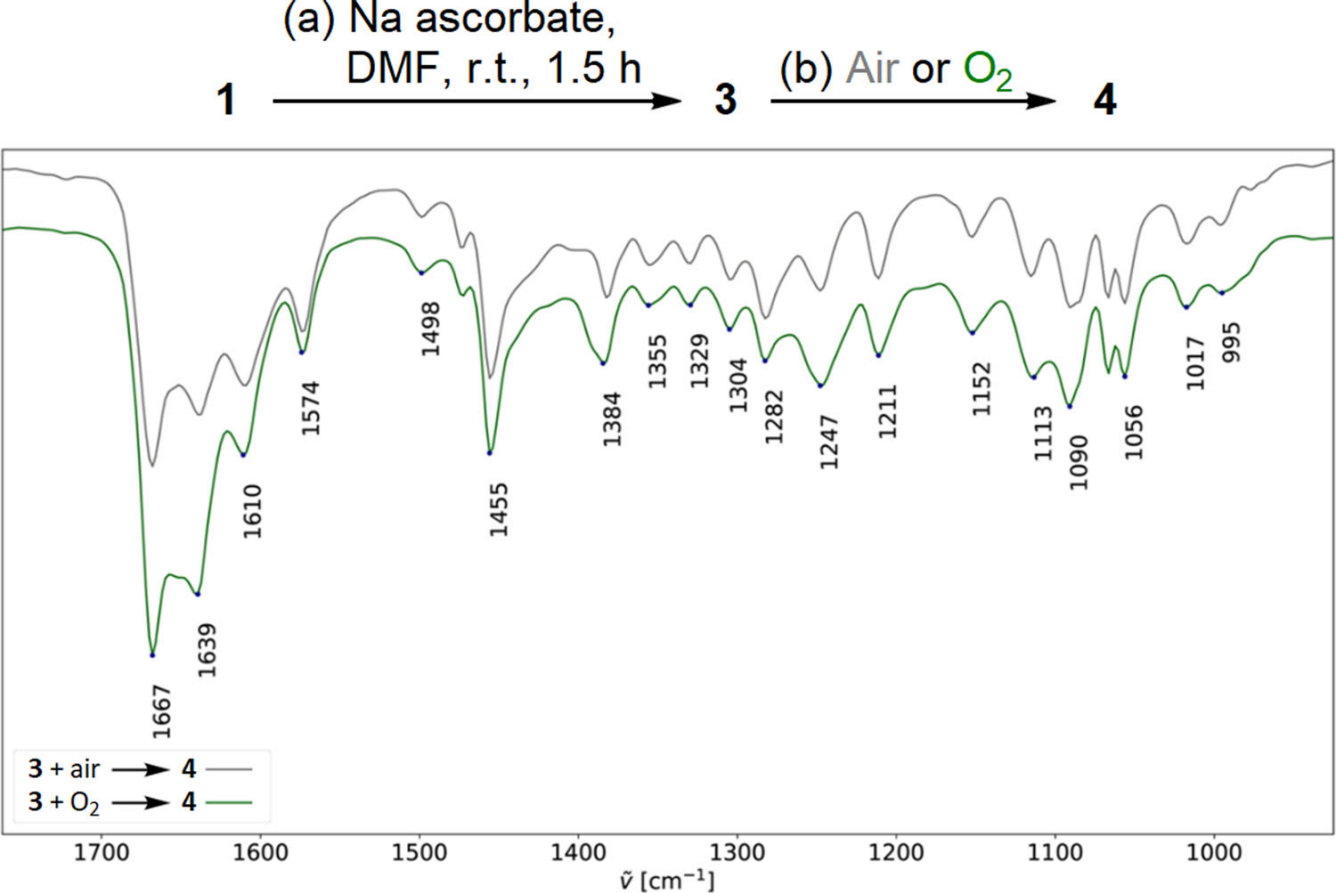
Formation of the oxalate complex 4 from 3 requires O_2_ and does not require CO_2_. FTIR spectra for the products obtained when **3** (prepared in situ from **1** and ascorbate) was exposed to air (grey) or pure O_2_ (green) are identical (details are given in the ESI).

Complex **4** was even obtained from a mixture of Cu(II) complex **1** and DHA in air, demonstrating that Cu(I) is not required for oxalate formation.

The Cu(I) complex [Cu_2_(*m*-xpt)_2_]^2+^ can also be prepared without ascorbate or dehydroascorbic acid, for example, by reaction of Cu(BF_4_)_2_ with Cu foil in DMF in the presence of *m*-xpt. This yellow solution of [Cu_2_(*m*-xpt)_2_](BF_4_)_2_ (**3a**, identical to **3** except for the counterion) (a) does not react with CO_2_; (b) reacts with air to produce the new trinuclear Cu(II) carbonate complex [Cu_3_(*m*-xpt)_3_(*μ*_3_-CO_3_)](BF_4_)_4_ (**6**); and (c) can be converted to the oxalate complex [Cu_2_(*m*-xpt)_2_(*μ*-C_2_O_4_)](BF_4_)_2_ (**4b**) by reaction with air or O_2_, but only if DHA is added. These observations are also in accordance with ascorbate being the source of oxalate. The structures of **6** and **4b** were determined by X-ray analysis (see Supplementary Fig. 13).

In the previous publication, an isotope labelling experiment was conducted by treating in situ generated **3** with ^13^CO_2_. In mass spectrometry experiments performed on **4** at that time, we did not observe signals attributable to oxalate-containing product ions; however, its FTIR spectrum appeared to show a shift of Δ*ṽ*_CO_ = −19 cm^−1^. Since this shift was only half the expected magnitude, we re-performed the labelling studies. In the new experiments, treatment of in situ generated **3** with ^13^CO_2_–O_2_ (1:1) produced only unlabelled **4**, whose ESI-MS shows a monoisotopic ion at 1147.1321 amu for [Cu_2_(*m*-xpt)_2_(μ-C_2_O_4_)](PF_6_)^+^ (see Supplementary Fig. 9). The ^13^C-labelled oxalate complex **4**-^13^C_2_ was obtained, for reference, by reaction of the starting complex **1** with (Bu_4_N)_2_(^13^C_2_O_4_); monoisotopic ion 1149.1373 amu. This analysis clearly demonstrates that oxalate does not arise from CO_2_ reduction. FTIR spectra of the new products show Δ*ṽ*_CO_ = −39 cm^−1^, close to the expected value (see Supplementary Fig. 8). A similar value for Δ*ṽ*_CO_ is also estimated based on DFT calculations; detailed results are given in the ESI.

In the previous publication, the IR absorption at ca. 1670 cm^−1^ in **4** was assigned to the oxalate C–O stretching vibration. However, as demonstrated in Supplementary Fig. 14, this absorption is caused by co-crystallized DMF in **4** (*ṽ*_CO_ for the bound oxalate is 1639 cm^−1^). In the previous experiment with ^13^CO_2_, **4** appeared to show an absorption at 1650 cm^−1^; we now know that this sample did not contain ^13^C_2_O_4_^2−^. The spectra in Supplementary Fig. 7 suggest that different samples of **4** may show varying absorption in the 1670–1640 cm^−1^ region. This variability may have led to the incorrect assignment of an apparent ^13^C shift in the previous work.

Due to this complexity of the IR spectra, we searched for additional experimental evidence for the formation of oxalate from the reaction under O_2_ atmosphere. We repeated the previously described oxalate removal by treatment with aqueous HNO_3_ (ref. ^16^), but we could not detect the expected H_2_C_2_O_4_ by ^13^C NMR spectroscopy. Therefore, we adapted a procedure which was utilised for the isolation of Na_2_C_2_O_4_ from similar Cu oxalate complexes^33^. We used this procedure to isolate Na_2_C_2_O_4_ (verified by ^13^C NMR spectroscopy), from samples of **4** obtained by reaction of in situ generated **3** with (a) air (i.e. O_2_ + CO_2_; Supplementary Fig. 18), and (b) pure O_2_ (i.e. without CO_2_; Supplementary Fig. 16). In the latter case, the isolation of Na_2_C_2_O_4_ from **4** was conducted under argon, so the isolated oxalate could not be formed by any reaction requiring CO_2_.

In summary, we have demonstrated that the Cu complex reported in the previous communication does not form oxalate via CO_2_ reduction. Instead, oxalate forms by oxidative degradation of ascorbate. This was finally evidenced by the reaction conducted under an atmosphere of O_2_, giving rise to the same oxalate complex described earlier (ref.^16^) from which sodium oxalate was removed and identified by NMR spectroscopy. In addition, the same product was obtained from reactions of the Cu(I) complex [Cu_2_(*m*-xpt)_2_]^2+^ with O_2_ or air in the presence of DHA. In experiments with [Cu_2_(*m*-xpt)_2_]^2+^ under ^13^CO_2_ + O_2_, ^13^C was not incorporated into the oxalate product. In contrast, a new trinuclear Cu(II) carbonate complex, [Cu_3_(*m*-xpt)_3_(*μ*-CO_3_)]^4+^, has been isolated, when [Cu_2_(*m*-xpt)_2_]^2+^ was treated with CO_2_ and O_2_ in the absence of sodium ascorbate or DHA. Since reproducibility is not always given for challenging transformations, such as the reductive coupling of CO_2_^34^, this report clearly highlights the importance of further mechanistic investigations on previously published systems.

## Supplementary information

Supplementary Information

## Data Availability

Accession codes: The X-ray crystallographic data for structures reported in this article have been deposited at the Cambridge Crystallographic Data Centre (CCDC), under deposition numbers CCDC 1976241 (**4b**) and 1976240 (**6**). These data can be obtained free of charge from the Cambridge Crystallographic Data Centre via https://www.ccdc.cam.ac.uk/structures/. Other data are available from the authors.
