# Supplementary material for: Oxalate production via oxidation of ascorbate rather than reduction of carbon dioxide
Source: Nat Commun. 2021 Mar 25;12:1997. doi: 10.1038/s41467-021-21817-w (PMC7994792; doi:10.1038/s41467-021-21817-w)
Supplement: Supplementary file 1 — Supplementary Information [file 41467_2021_21817_MOESM1_ESM.pdf]

Oxalate production via oxidation of ascorbate rather than reduction of carbon dioxide

Electronic Supplementary Information

Arising from: Pokharel et al. *Nature Commun.* https://doi.org/10.1038/ncomms6883 (2014)

Supplementary Methods S-3

General S-3

Summary of procedures and results S-4

Treatment of *in situ* generated $[Cu_2(m\text{-xpt})_2](PF_6)_2$ (**3**) with CO_2, followed by reaction with air S-5

Reaction of *in situ* generated $[Cu_2(m\text{-xpt})_2](PF_6)_2$ (**3**) with O_2 .... S-7

Reaction of *in situ* generated $[Cu_2(m\text{-xpt})_2](PF_6)_2$ (**3**) with O_2 in the presence of $^{13}CO_2$ S-10

Reaction of $[Cu_2(m\text{-xpt})_2(NO_3)_2](PF_6)_2$ (**1**) and dehydroascorbic acid (DHA) with air S-10

Preparation of authentic $[Cu^{II}_2(m\text{-xpt})_2(^{13}C_2O_4)](PF_6)_2$ (**4**-$^{13}C_2$) *via* $(Bu_4N)_2{}^{13}C_2O_4$ S-11

Synthesis of $[Cu_2(m\text{-xpt})_2](PF_6)_2$ (**3**)[4] S-14

Preparation of $[Cu^I_2(m\text{-xpt})_2]^{2+}$ by other routes, and its reactivity S-16

$[Cu^I_2(m\text{-xpt})_2](BF_4)_2$ (**3a**) in DMF solution. S-16

$[Cu_3(m\text{-xpt})_3(\mu_3\text{-}CO_3)](BF_4)_4 \cdot 2.5DMF$ (**6**). S-17

Reaction of $[Cu^I_2(m\text{-xpt})_2](BF_4)_2$ (**3a**) with air in the presence of DHA. S-17

Comparison of the FTIR spectra of Cu(II) complex **1**, Cu(I) complex **3**, Cu(II) oxalate complex **4**, and DMF S-19

Isolation of oxalate from complex **4** S-19

Isolation of oxalate from complex **4** prepared by reaction of *in situ* generated **3** with O_2 .... S-19

Isolation of oxalate from complex **4** prepared by reaction of *in situ* generated **3** with air S-21

Isolation of oxalate from complex **4a** prepared from **1** and $(Bu_4N)_2C_2O_4$ S-24

Quantum chemical (DFT) calculations S-27

X-ray crystallography S-28

Common ascorbate oxidation products...S-30

Supplementary References S-31

Supplementary Methods

General

Unless stated otherwise, reactions were conducted under an atmosphere of Ar or N_2. Commercially available reagents were purchased from Alfa Aesar, BeanTown Chemicals, ICON Isotopes, Sigma-Aldrich, and TCI and were used without further purification. DMSO-d_6 was dried over activated molecular sieves (3 Å) and degassed by passing a stream of Ar through the solution for a minimum of 45 min. H_2O and D_2O were degassed using a stream of Ar. Anhydrous CH_2Cl_2, diethyl ether, toluene, and DMF were obtained from an Inert Technologies solvent purification system (passing through a column of activated alumina), degassed and stored under Ar (CH_2Cl_2 and toluene stored over activated 3 Å molecular sieves). $(Bu_4N)_2(C_2O_4)$[1], *m*-xpt[2,3], $[Cu_2(m\text{-xpt})_2(NO_3)_2](PF_6)_2$ (**1**)[4], and $[Cu_2(m\text{-xpt})_2](PF_6)_2$ (**3**)[4] were prepared according to literature procedures.

NMR spectra were recorded on Bruker 300 MHz (Avance 300, Fourier 300) and 400 MHz (Avance 400) spectrometers. Secondary referencing of ^{1}H and $^{13}C\{^{1}H\}$ NMR spectra was performed utilizing the solvent residual signal[5]. $^{31}P\{^{1}H\}$ NMR spectra are reported relative to an external 85% H_3PO_4 standard. FTIR spectra (ATR, diamond) were recorded on a Tensor 27 or Alpha P FTIR spectrometer (Bruker). Electrospray ionization mass spectra (ESI-MS) were measured on an Agilent 6210 instrument. UV/Vis spectra were recorded on a Specord S600 diode array spectrometer (Analytik Jena) or an Aviv 14DS spectrometer (Aviv Biomedical). (Note: the wavelengths reported in the previous publication[4] were too high by 20 nm, due to the inadvertent use of the wrong instrument calibration file in data collection. The correct λ_{max} values in DMF solution are as follows: d-d band in **1**, 720 nm; MLCT band in **3**, 364 nm; d-d band in **4**, 731 nm.)

.

Summary of procedures and results

Supplementary Table 1. Summary of procedures and results

**Reactants etc.**	**Gas(es) used**	**Results (products, comments)**	**Page**
$[Cu_2(m\text{-xpt})_2](PF_6)_2$ (**3**) + dehydroascorbic acid (DHA) *in situ*[a]	(a) CO_2;<br>(b) O_2 + CO_2 (air)	(a) n. r.;<br>(b) **4**	S-5
**3** + DHA *in situ*	O_2 (no CO_2)	**4**	S-7
**3** + DHA *in situ*	$^{13}CO_2$ + O_2 (~1:1)	**4**	S-10
**1** + added DHA	O_2 + CO_2 (air)	**4**	S-10
$[Cu_2(m\text{-xpt})_2(ONO_2)_2](PF_6)_2$ (**1**) + $(Bu_4N)_2(^{13}C_2O_4)$	N_2	**4**-$^{13}C_2$	S-11
Synthesis of **3**		Solid **3** contains DMF	S-14
$[Cu_2(m\text{-xpt})_2]^{2+}$	(a) CO_2;<br>(b) O_2 + CO_2 (air)	(a) n. r.;<br>(b) **6**[b]	S-16
$[Cu_2(m\text{-xpt})_2](BF_4)_2$ (**3a**) + added DHA	(a) CO_2;<br>(b) O_2 + CO_2 (air)	(a) n. r.;<br>(b) **4b**[c]	S-17
**4** + NaOH(aq)		$Na_2C_2O_4$	S-19
DFT calculations		Calculated values of $\tilde{\nu}_{asym}$(CO) for **4**	S-27

[a]When **3** is prepared from **1** by reaction with ascorbate, DHA is also present in the solution. [b]**6** = $[Cu_3(m\text{-xpt})_3(\mu_3\text{-}CO_3)]$ $(BF_4)_4$·2.5DMF. [c]**4b** = $[Cu_2(m\text{-xpt})_2(\mu\text{-}C_2O_4)](BF_4)_2$·2DMF.

Treatment of *in situ* generated $[Cu_2(m\text{-xpt})_2](PF_6)_2$ (3) with CO_2, followed by reaction with air

To $[Cu_2(m\text{-xpt})_2(NO_3)_2](PF_6)_2$ (**1**) (49.9 mg, 37.5 µmol, 1.00 equiv.) and sodium ascorbate (10.8 mg, 54.5 µmol, 1.45 equiv.) under an atmosphere of Ar was added dry DMF (4.0 mL). The green solution was stirred at r.t. for 1 h and the atmosphere subsequently exchanged to CO_2 by purging the Schlenk flask with CO_2 for 2 min. The flask was sealed under a CO_2 overpressure and the reaction mixture stirred at room temperature for 6 days. There was no observable color change on stirring under CO_2 for 6 days (see Supplementary Figure 1). A sample of the yellow solution (0.5 mL) was transferred into a UV/Vis cuvette equipped with a Teflon-coated screw cap septum, diluted with dry DMF (1.0 mL) to a concentration of 3.1 mM and the hole in the septum sealed by molten Parafilm. After recording the UV/Vis spectrum of the reaction mixture, air (4 mL) was introduced into the UV/Vis cuvette and the oxidation followed by UV/Vis spectroscopy (Supplementary Figure 2). Air (1 mL at first, followed by 12 mL after 7 days) was also introduced to the main reaction mixture and stirring continued for a total of 11 days (the distinct color change from yellow to green is depicted in Supplementary Figure 1). After removal of the volatiles *in vacuo*,

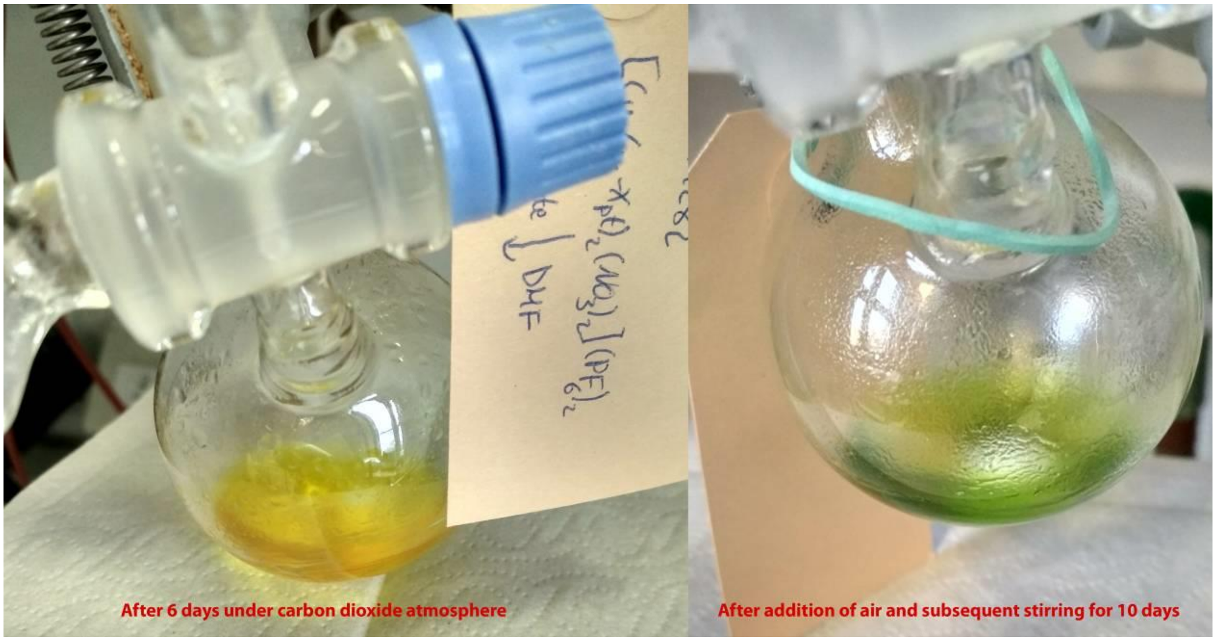

Supplementary Figure 1 | *In situ* prepared complex 3 under CO_2 over 6 days (left), and after reaction with air and stirring for another 10 days (right).

the green solid residue was dissolved in DMF, transferred into a vial in air, and left for slow evaporation of the solvent. This produced green crystals, which were shown to be identical to **4** by FTIR spectroscopy (Supplementary Figure 3).

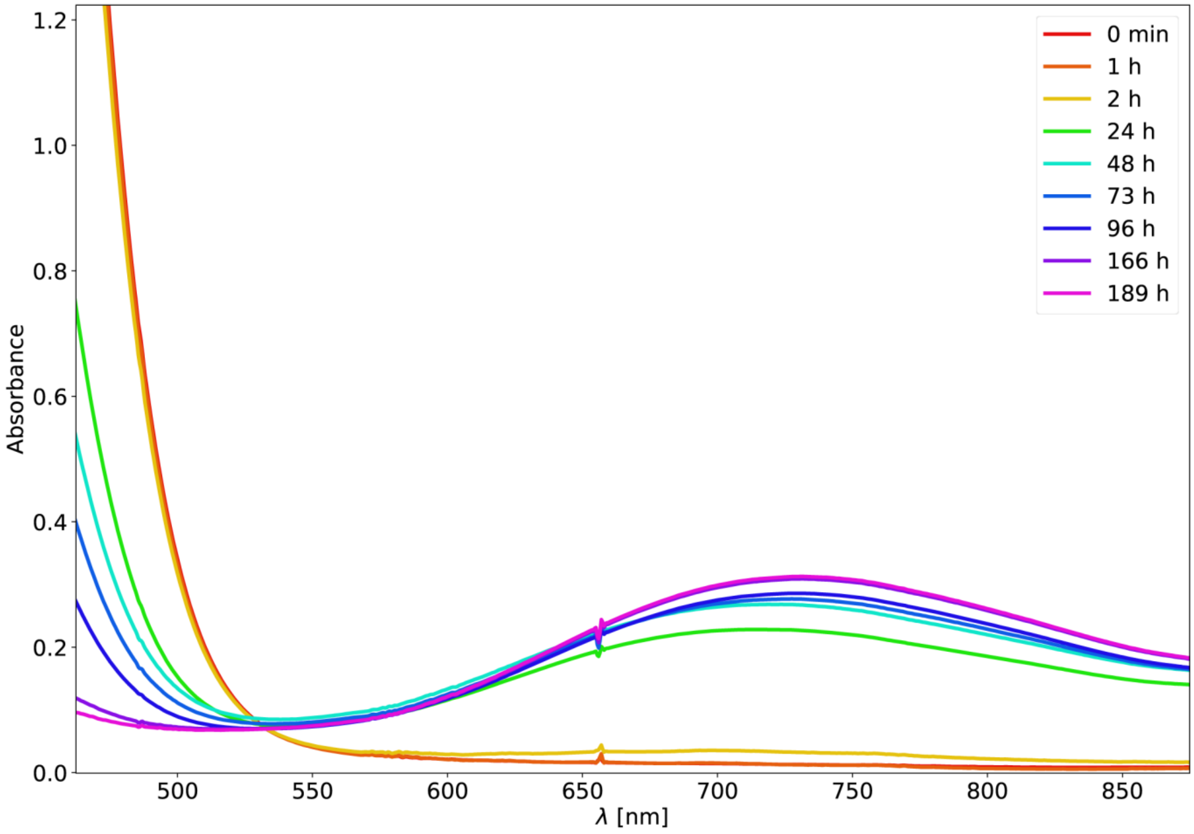

Supplementary Figure 2 | Spectral changes during the reaction of the Cu(I) complex with air. Solution of *in situ* generated [Cu_2(*m*-xpt)$_2$](PF_6)$_2$ (**3**) starting from complex **1** and sodium ascorbate in DMF reacted with CO_2 for 6 days, diluted with DMF to 3.1 mM (entry 0 min) and subsequently exposed to air (followed over the time period from 1 h to 189 h).

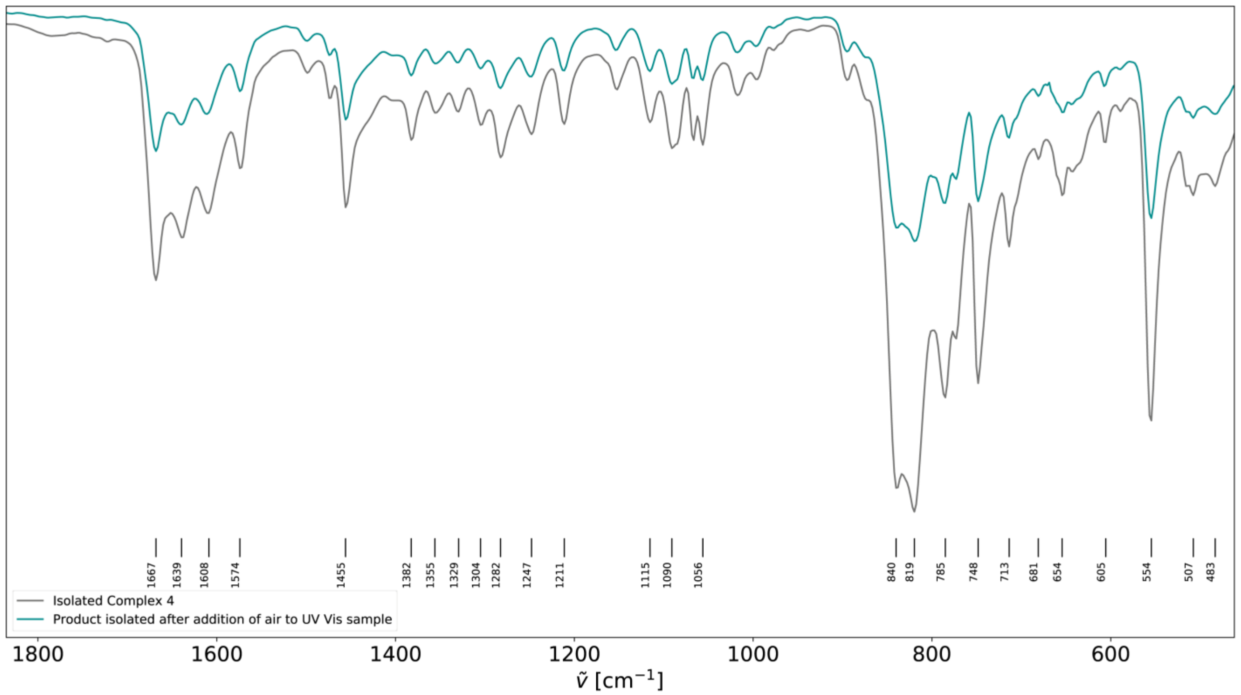

Supplementary Figure 3 | FTIR spectra of the oxalate complex 4 and the product obtained from the UV/Vis sample. Isolated complex **4** (grey spectrum) was prepared according to the previously reported procedure *via* reaction of the *in situ* generated Cu(I) complex **3** with air.[4] A sample from the reaction depicted in Supplementary Figure 1 was left to evaporate to give the blue-green FTIR spectrum.

Reaction of *in situ* generated $[Cu_2(m\text{-xpt})_2](PF_6)_2$ (3) with O_2

To complex **1** (110.2 mg, 82.9 µmol, 1.00 equiv.) and sodium ascorbate (25.1 mg, 127 µmol, 1.53 equiv.) under an atmosphere of Ar was added dry DMF (11 mL) and the resulting green solution was stirred at r.t. for 1.5 h. The atmosphere was exchanged for O_2 by bubbling O_2 *via* a balloon through the yellow solution (Supplementary Figure 4). The Schlenk tube was closed and the reaction mixture stirred at room temperature for five days, during which the color changed from yellow to green/blue-green (Supplementary Figure 5). Volatiles were removed *in vacuo*, yielding a green/yellow solid, which was analyzed by FTIR spectroscopy (Figure 1 & Supplementary Figure 6) and subsequently submitted to oxalate removal by treatment with aqueous NaOH. The same procedure was utilized with a prolonged reaction time of seven days under O_2 atmosphere, yielding essentially identical FTIR spectra (Supplementary Figure 7).

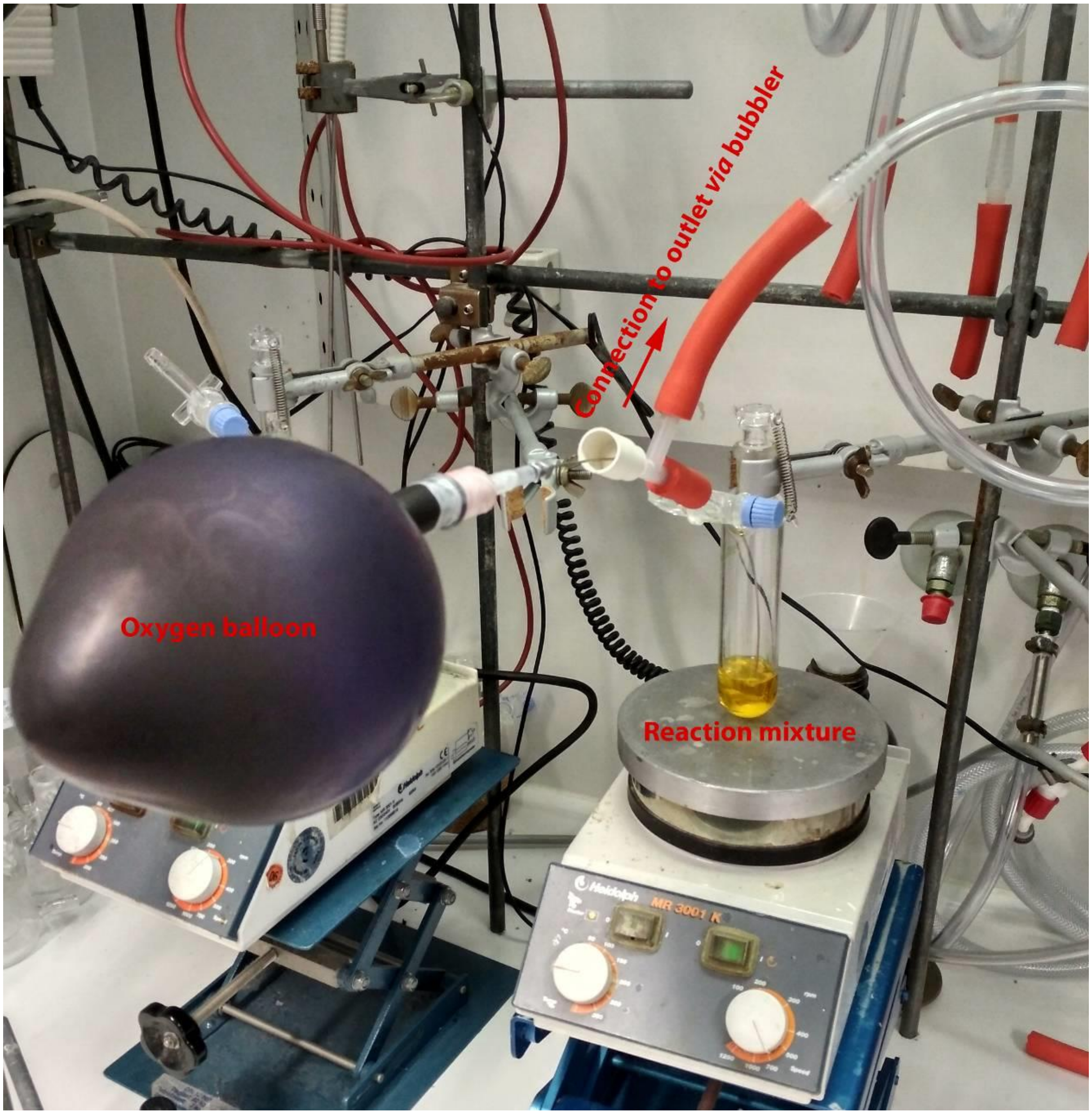

Supplementary Figure 4 | Setup for the reaction of *in situ* prepared complex 3 with O_2 under exclusion of air/CO_2.

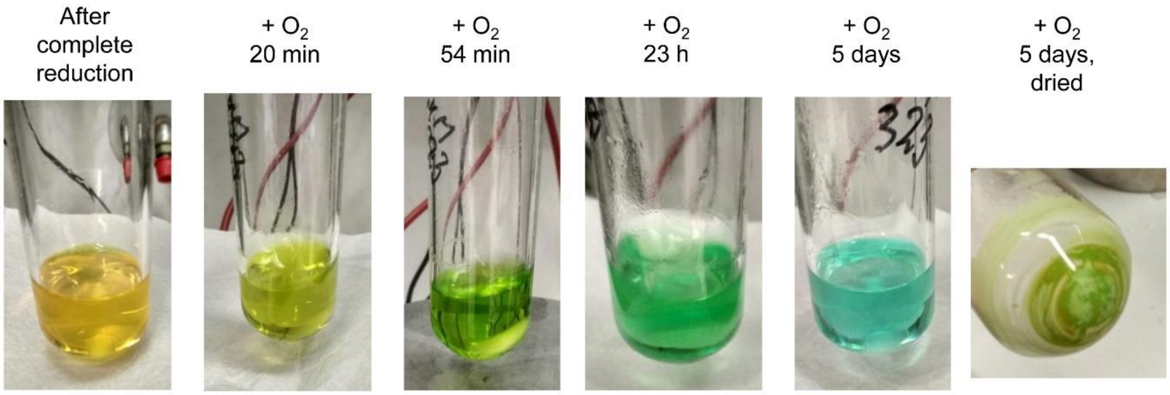

Supplementary Figure 5 | Time-dependent color change of the reaction of *in situ* prepared Cu(I) complex 3 with O_2.

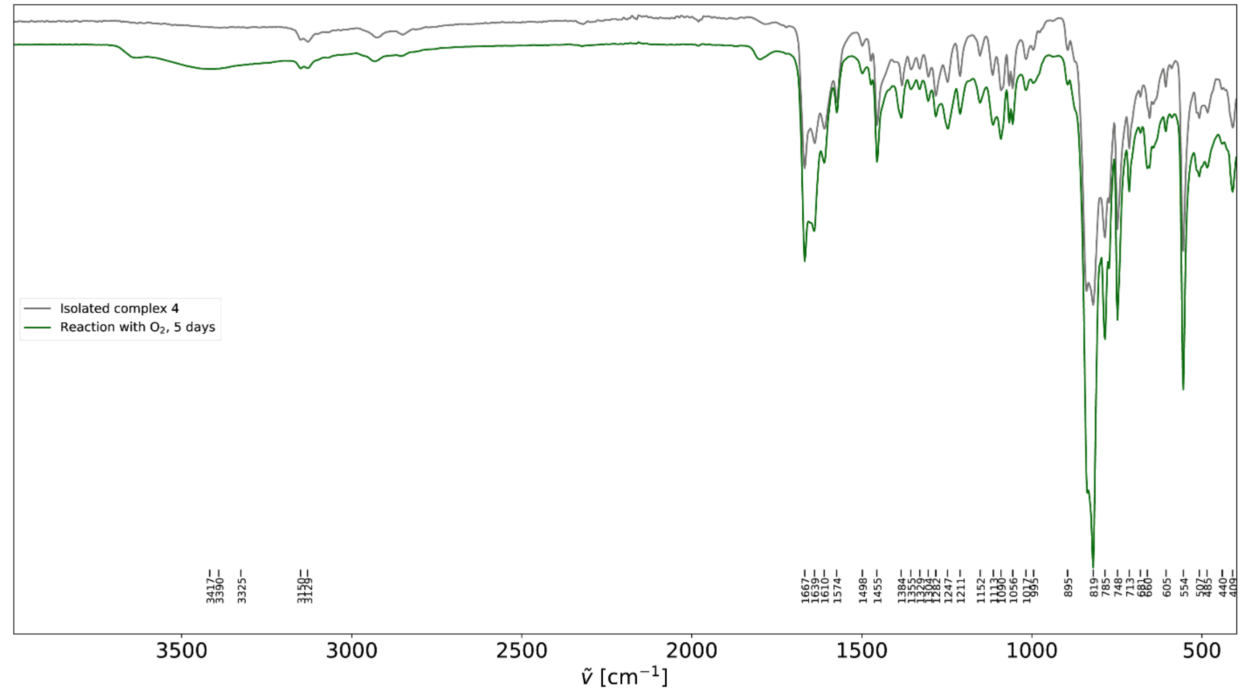

Supplementary Figure 6 | FTIR spectra of the isolated complex 4 (prepared by reaction with air, same sample as gray trace in Supplementary Figure 3) and the product isolated from the reaction with O_2 under exclusion of air/CO_2 after 5 days.

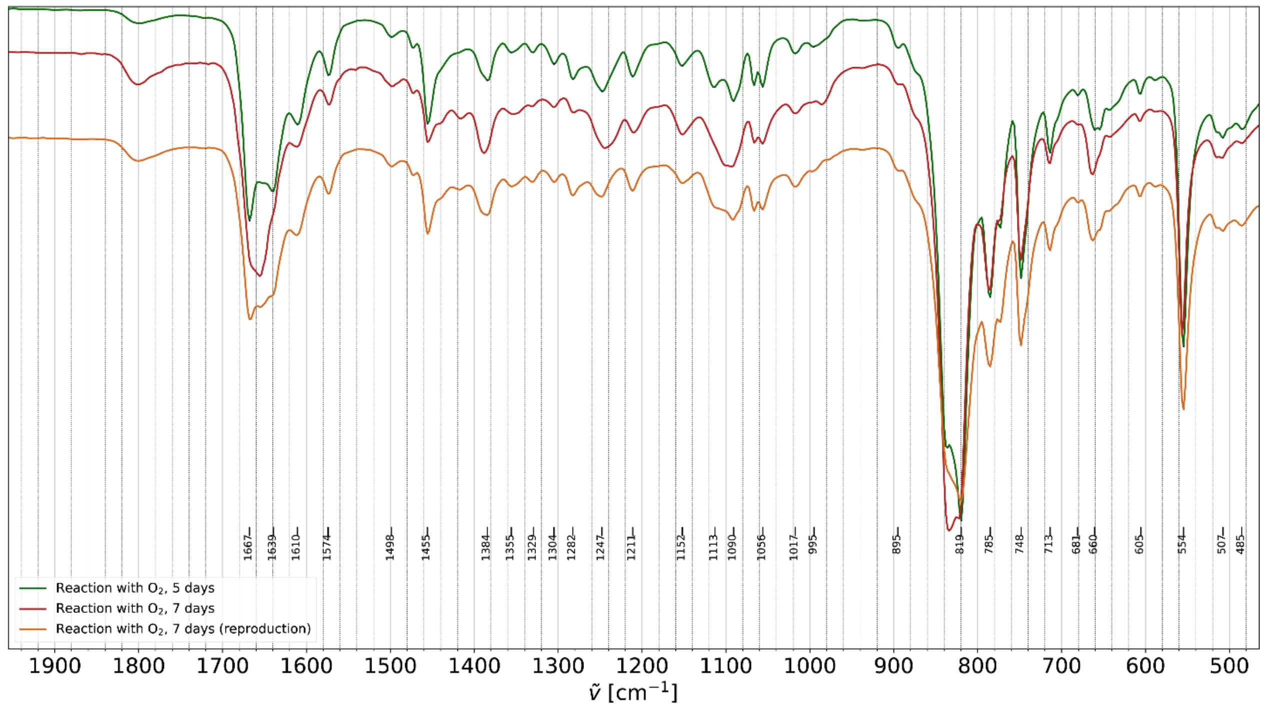

Supplementary Figure 7 | Comparison of the FTIR spectra of 4, obtained from the reaction of *in situ* prepared complex 3 with O_2 after 5 days (2 trials) and 7 days.

Reaction of *in situ* generated $[Cu_2(m\text{-xpt})_2](PF_6)_2$ (3) with O_2 in the presence of $^{13}CO_2$

A similar DMF solution of *in situ* generated $[Cu_2(m\text{-xpt})_2](PF_6)_2$ (**3**) was exposed to a mixture of O_2 and $^{13}CO_2$ (~1:1). This procedure also generated the oxalate complex **4**, which showed no evidence of ^{13}C labeling; see FTIR and ESI-MS data in Supplementary Figure 8 and Supplementary Figure 9. The band at 1668 cm^{-1} in **4** was previously assigned as $\tilde{\nu}_{CO}$ of oxalate 4. However, since the peak shows negligible shift between **4** and **4**-$^{13}C_2$ (see Supplementary Figure 8), we now assign it to co-crystallized solvent DMF.

Reaction of $[Cu_2(m\text{-xpt})_2(NO_3)_2](PF_6)_2$ (1) and dehydroascorbic acid (DHA) with air

Equimolar amounts of $[Cu_2(m\text{-xpt})_2(NO_3)_2](PF_6)_2$ (**1**) and DHA were dissolved in DMF, and the solution stirred in air for 7 days. Crystals of **4** were deposited by vapor diffusion of ether into this solution. Thus, reduction of Cu(II) to Cu(I) is not required for oxalate to be generated and incorporated into the host complex.

Preparation of authentic [Cu^{II}_2(*m*-xpt)$_2$($^{13}C_2O_4$)](PF_6)$_2$ (4-$^{13}C_2$) *via* (Bu_4N)$_2$$^{13}C_2O_4$

$H_2{}^{13}C_2O_4$ (4.8 mg, 0.037 mmol) and Bu_4NOH (62.4 mg of 1 M solution in methanol, density 0.83 g/mL; 0.075 mmol) were mixed in 20 mL methanol under N_2, and the solution was stirred under N_2 for ca. 16 h. The solvent was evaporated to give $(Bu_4N)_2{}^{13}C_2O_4$ as a white solid. N_2-purged DMF (2 mL) was added to this solid, and the resulting solution added dropwise to **1** (49.6 mg, 0.0373 mmol, in 5 mL of N_2-purged DMF), with stirring. The color of the solution gradually changed from blue to green. Addition was stopped when the first sign of permanent turbidity was observed. At this point the solution was stirred for one additional hour under N_2, and then set up for crystallization by ether vapor diffusion. After a week, green crystals of **4**-$^{13}C_2$ ([Cu_2(*m*-xpt)$_2$($^{13}C_2O_4$)](PF_6)$_2$) were collected, washed with ether, and dried in air (25.7 mg, yield 53 %). See FTIR and ESI-MS data in Supplementary Figure 8 and Supplementary Figure 9.

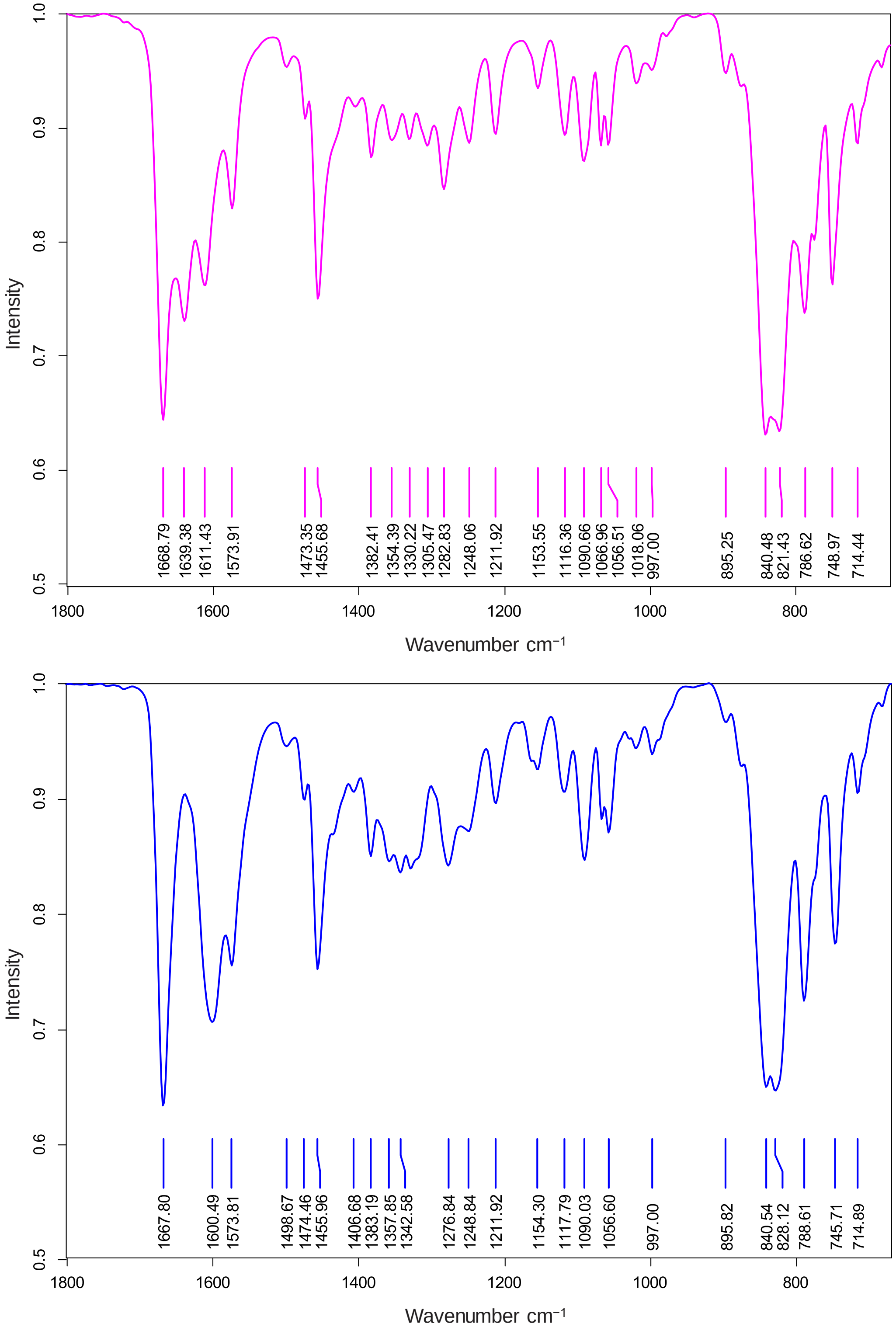

Supplementary Figure 8 | FTIR spectra of 4 and 4-$^{13}C_2$. Top (magenta), unlabeled, prepared from **1** by ascorbate reduction, followed by exposure to $^{13}CO_2$ + O_2. Bottom (blue), ^{13}C-labeled, prepared from **1** by treatment with $(Bu_4N)_2(^{13}C_2O_4)$.

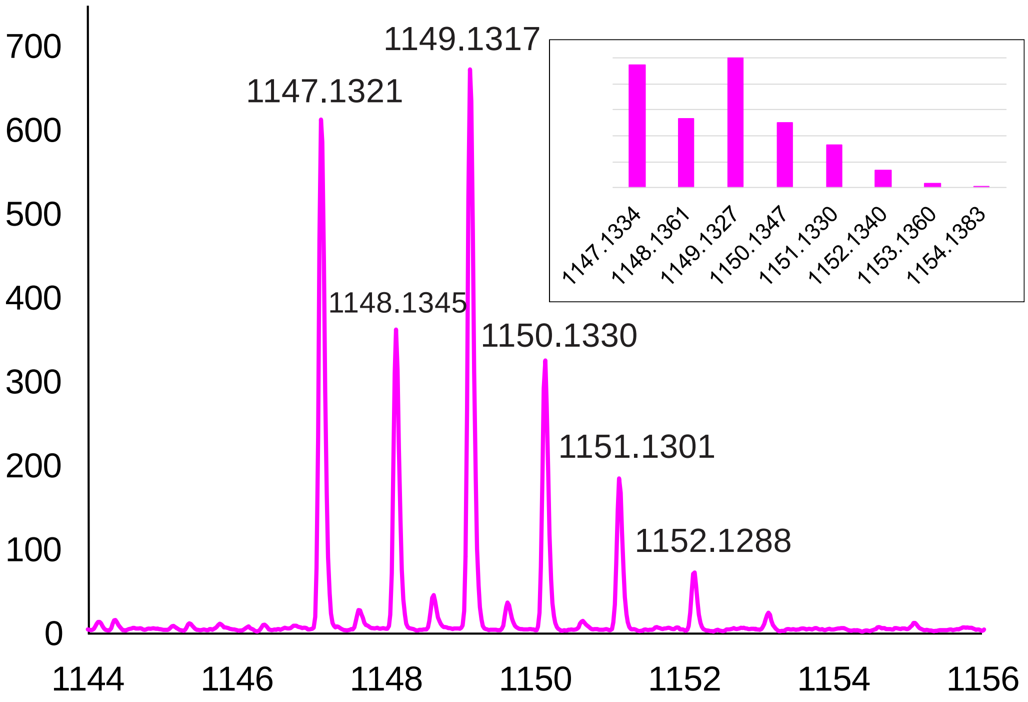

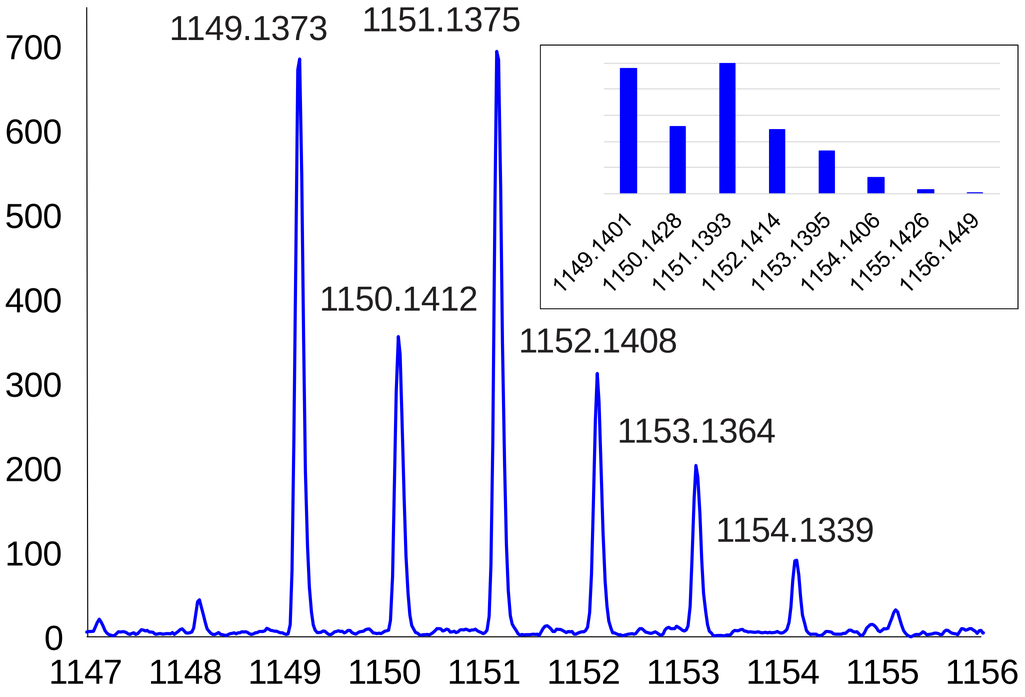

Supplementary Figure 9 | ESI mass spectra, [Cu_2(*m*-xpt)$_2$(*μ*-C_2O_4)](PF_6)$^+$ peak, from 4 and 4-$^{13}C_2$. Top (magenta), unlabeled, prepared from **1** by ascorbate reduction, followed by exposure to $^{13}CO_2$ + O_2. Bottom (blue), ^{13}C-labeled, prepared from **1** by treatment with $(Bu_4N)_2(^{13}C_2O_4)$. Insets are calculated isotopic masses and relative intensities.

Synthesis of $[Cu_2(m\text{-xpt})_2](PF_6)_2$ (3)[4]

To complex **1** (111.0 mg, 83.5 μmol, 1.00 equiv.) and sodium ascorbate (16.8 mg, 84.8 μmol, 1.02 equiv.) under an atmosphere of Ar was added dry DMF (5.5 mL). The green solution was stirred at r.t. for 1 h, yielding a yellow solution. Slow vapor diffusion of dry Et_2O (22 mL) into the yellow DMF solution at −32 °C resulted in a yellow solid precipitate, which was filtered off using a Teflon cannula equipped with a glass microfiber filter. Washing with dry Et_2O (4 mL) and drying of the resulting yellow solid *in vacuo* gave **3** (94.9 mg), which still contained DMF as evident from NMR spectroscopic analysis (Supplementary Figure 10).

For a comparison of IR spectra of **1**, **3**, **4**, and DMF, see Supplementary Figure 14.

^{1}H NMR (DMSO-d_6, 300 MHz): δ [ppm] = 9.18 (br, 4H), 8.45 (br, 4H), 8.11 (br, 8H), 7.48 (br, 10H), 7.33 (br, 2H), 5.78 (s, 8H).

^{31}P{^{1}H} NMR (DMSO-d_6, 122 MHz): δ [ppm] = −144.2 (sept, J = 711 Hz).

FTIR (neat): $\tilde{\nu}$ = 3099, 3016, 2930, 2864, 2808, 1661, 1607, 1569, 1449, 1388, 1360, 1343, 1320, 1256, 1237, 1205, 1158, 1090, 1055, 988, 832, 779, 743, 774, 660, 638, 611, 555, 512, 498 cm^{-1}.

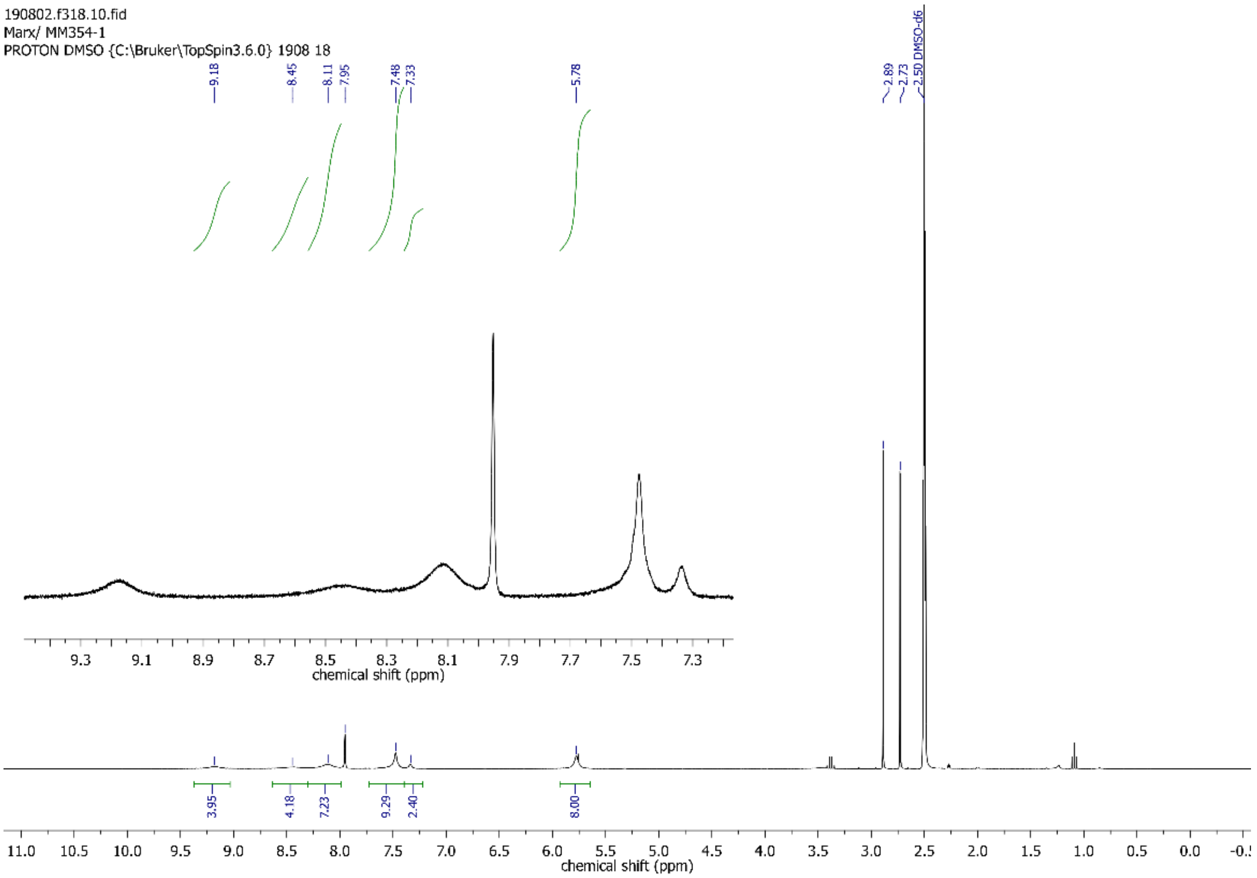

Supplementary Figure 10 | ^{1}H NMR spectrum (DMSO-d_6, 300 MHz) of the isolated Cu(I) complex 3.

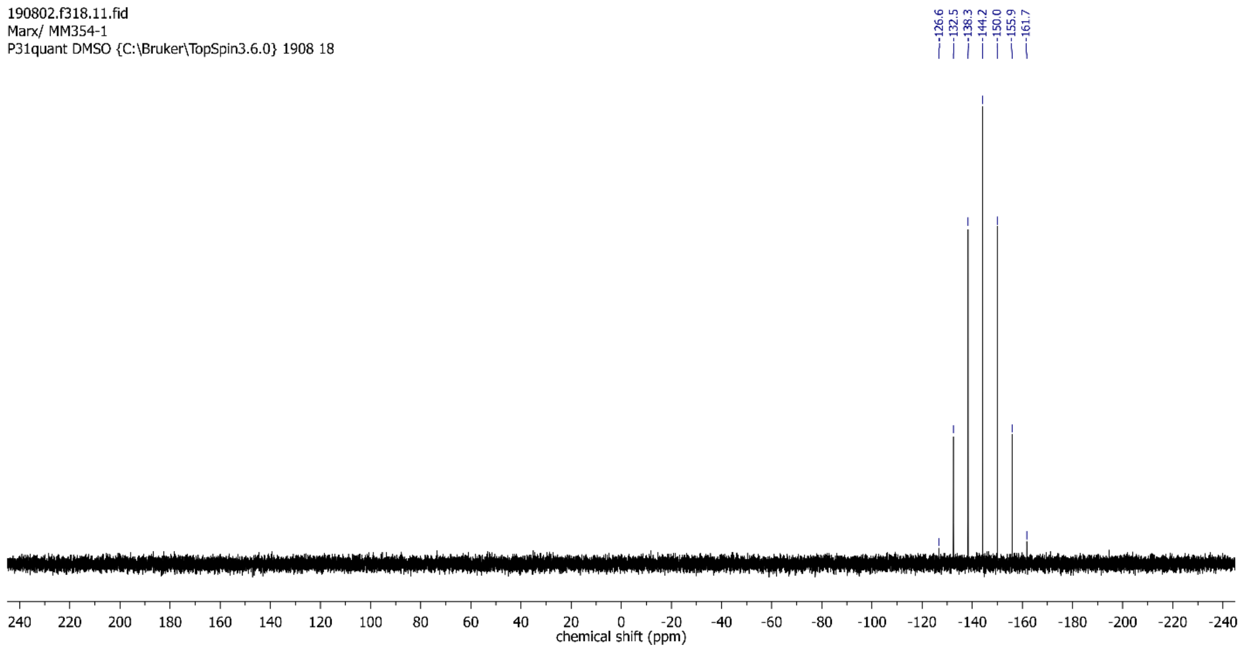

Supplementary Figure 11 | ^{31}P{^{1}H} NMR spectrum (DMSO-d_6, 122 MHz) of the isolated Cu(I) complex 3.

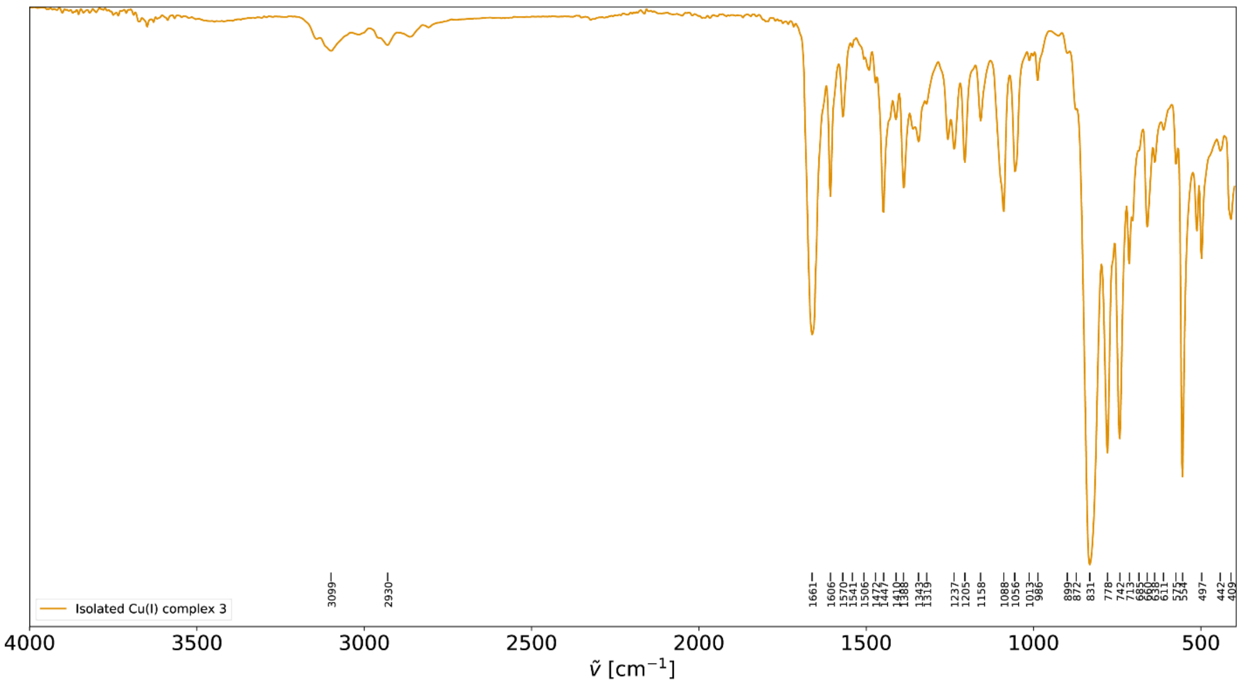

Supplementary Figure 12 | FTIR spectrum of the isolated Cu(I) complex 3.

Preparation of $[Cu^{I}_2(m\text{-xpt})_2]^{2+}$ by other routes, and its reactivity

Solutions of $[Cu^{I}_2(m\text{-xpt})_2]^{2+}$ containing no ascorbate or dehydroascorbic acid were prepared by three methods: (i) from solid **3**, in fresh DMF; (ii) from $[Cu(CH_3CN)_4](PF_6)$ in DMF; and (iii) by comproportionation. For route (iii), reaction of $Cu(BF_4)_2$ with copper foil in DMF produced mostly Cu(I), but UV-vis spectra of these solutions indicated that some Cu(II) remained. This is not surprising, given the relative stability of Cu(I) and Cu(II) in different solvents[6]. However, when a stoichiometric amount of *m*-xpt was added to the mixture, reaction to produce Cu(I) went essentially to completion.

$[Cu^{I}_2(m\text{-xpt})_2](BF_4)_2$ (3a) in DMF solution.

Copper(II) tetrafluoroborate and *m*-xpt (in 1:2 ratio), and an excess of copper foil were added to a reaction flask sealed to a cuvette. The flask was evacuated for 30 minutes. Then, N_2-purged anhydrous DMF was added and the mixture was stirred under flowing N_2 for 3 hours to achieve complete reduction of Cu(II) to Cu(I). The resulting $[Cu^{I}_2(m\text{-xpt})_2](BF_4)_2$ solution was transferred to

a Schlenk flask *via* cannula under N_2 for subsequent experiments, to remove it from the unreacted Cu foil.

[Cu_3(*m*-xpt)$_3$(μ_3-CO_3)](BF_4)$_4$·2.5DMF (6).

A solution of [Cu^I_2(*m*-xpt)$_2$](BF_4)$_2$ (**3a**) in DMF was prepared as above, from Cu(BF_4)$_2$ (12.7 mg, 0.0535 mmol), *m*-xpt (42.2 mg, 0.107 mmol), excess copper foil (ca. 0.2 g) and 5 mL N_2-purged anhydrous DMF. This solution was stable under N_2 and CO_2. It was then exposed to air and stirred for 3 days, and used for crystallization by ether vapor diffusion. Blue plates of **6** were collected and analyzed by X-ray crystallography; see Supplementary Figure 13(a) and p. S-28. ESI-MS: m/z 1806.3013, 1806.2976 ([Cu_3(*m*-xpt)$_3$](BF_4)$_5^+$, calcd. 1806.2997).

Reaction of [Cu^I_2(*m*-xpt)$_2$](BF_4)$_2$ (3a) with air in the presence of DHA.

A solution of [Cu^I_2(*m*-xpt)$_2$](BF_4)$_2$ (**3a**) in DMF was prepared as above, from Cu(BF_4)$_2$ (20.7 mg, 0.0874 mmol), *m*-xpt (70.0 mg, 0.178 mmol), excess copper foil (ca. 0.2 g) and 5 mL N_2-purged anhydrous DMF. This solution was transferred *via* cannula to a flask containing an equimolar amount of DHA, under N_2, with stirring. This solution was stable under N_2 and CO_2. However, when it was left to evaporate in air over a period of days, green crystals of [Cu_2(*m*-xpt)$_2$(μ-C_2O_4)](BF_4)$_2$·2DMF (**4b**) formed. For X-ray analysis of **4b**, see Supplementary Figure 13(b) and p. S-28.

(a)

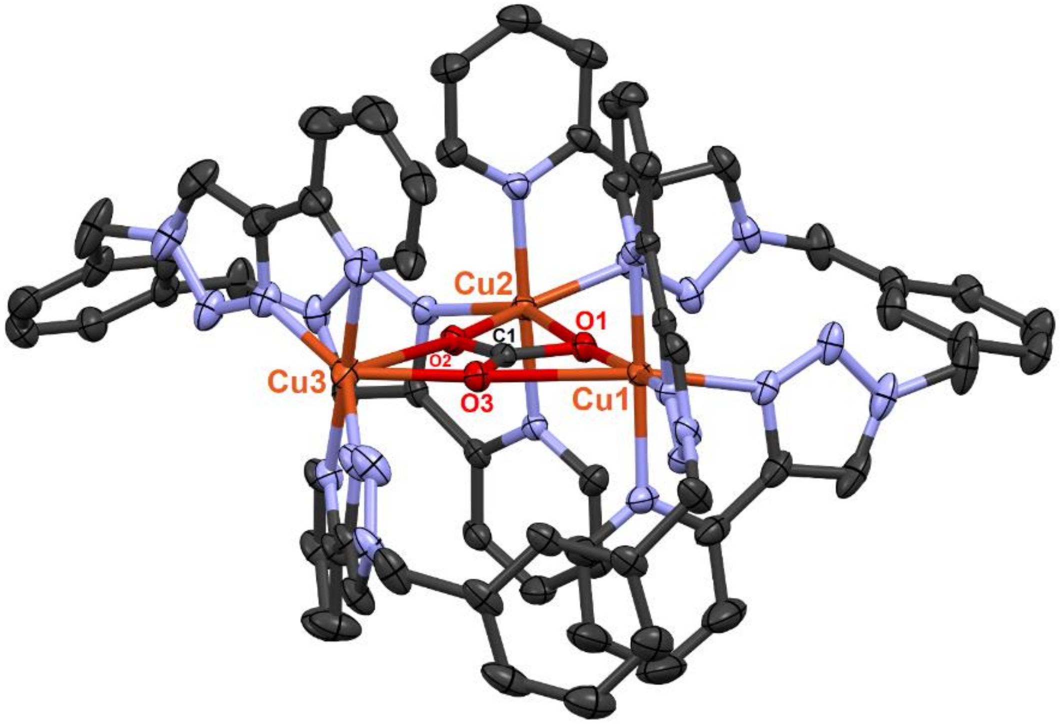

(b)

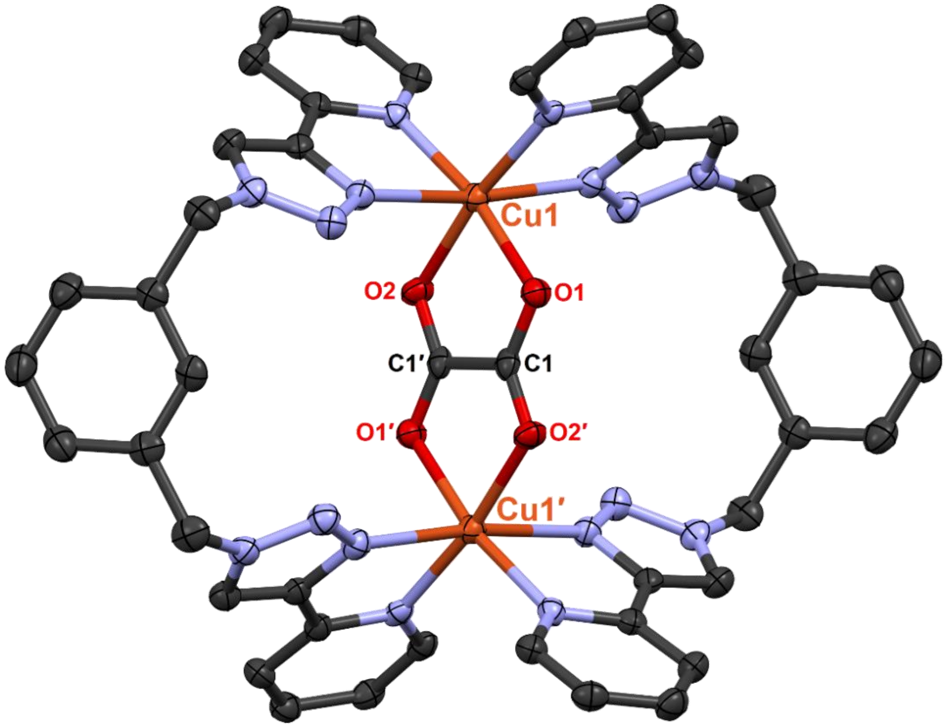

Supplementary Figure 13 | Crystal structure illustrations. (a) [Cu_3(*m*-xpt)$_3$(μ_3-CO_3)](BF_4)$_4$·2.5DMF (6). Cu1⋯Cu2 4.4903(8), Cu2⋯Cu3 4.9275(9), Cu3⋯Cu1 4.6737(5) Å. **(b) [Cu_2(*m*-xpt)$_2$(μ-C_2O_4)](BF_4)$_2$·2DMF (4b).** Cu1⋯Cu1′ 5.2123(6) Å. In both structures, anions, solvent molecules, and hydrogen atoms were omitted for clarity; ellipsoids are at the 50% probability level. For details, see X-ray crystallography section on p. S-28.

Comparison of the FTIR spectra of Cu(II) complex 1, Cu(I) complex 3, Cu(II) oxalate complex 4, and DMF

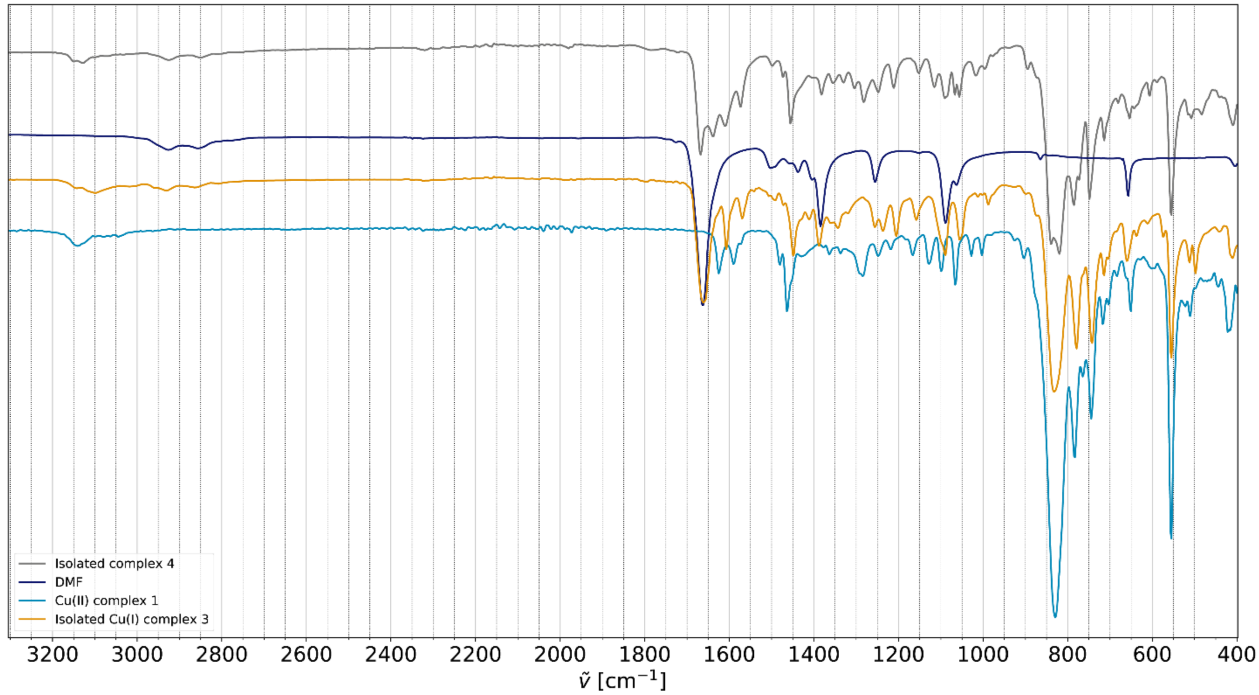

Supplementary Figure 14 | FTIR spectra of Cu(II) complex 1, Cu(I) complex 3, oxalate complex 4, and DMF.

The FTIR spectra of **1**, **3**, **4**, and DMF are similar; in particular, solids **3** and **4** contain DMF, as judged by the absorption at ca. 1668 cm^{-1}. This makes distinguishing these materials by their IR spectra challenging.

Isolation of oxalate from complex 4

Isolation of oxalate from complex 4 prepared by reaction of *in situ* generated 3 with O_2

The removal of oxalate was performed utilizing the procedure described on p. S-19. To the crude reaction mixture of the reaction with O_2 (containing the oxalate product **4**) was added dry toluene (3.6 mL) and degassed 1 M aqueous NaOH (1.8 mL, 1.80 mmol). The reaction mixture was stirred under an atmosphere of Ar for 17 h, yielding a yellow suspension. Volatiles were removed *in vacuo* and the yellow solid residue extracted with dry CH_2Cl_2 (4.0 mL). To the solid residue was added

degassed D_2O (1.0 mL), the yellow suspension transferred into a J. Young NMR tube under Ar and subsequently analysed by NMR spectroscopy (Supplementary Figure 15 and Supplementary Figure 16). To the same sample were added (after complete NMR spectroscopic analysis) $Na_2C_2O_4$ and sodium formate as internal standards for direct determination of the oxalate/formate ^{13}C resonances. Note: Sodium formate detected by NMR spectroscopy is formed by alkaline hydrolysis of co-crystallized DMF (as detected by FTIR spectroscopy, see Supplementary Figure 14).

^{1}H NMR (D_2O, 300 MHz): δ [ppm] = 8.40 (s, HCO_2Na), 5.41 (residual CH_2Cl_2).

^{13}C{^{1}H} NMR (D_2O, 75 MHz): δ [ppm] = 173.3 ($Na_2C_2O_4$), 171.1 (HCO_2Na).

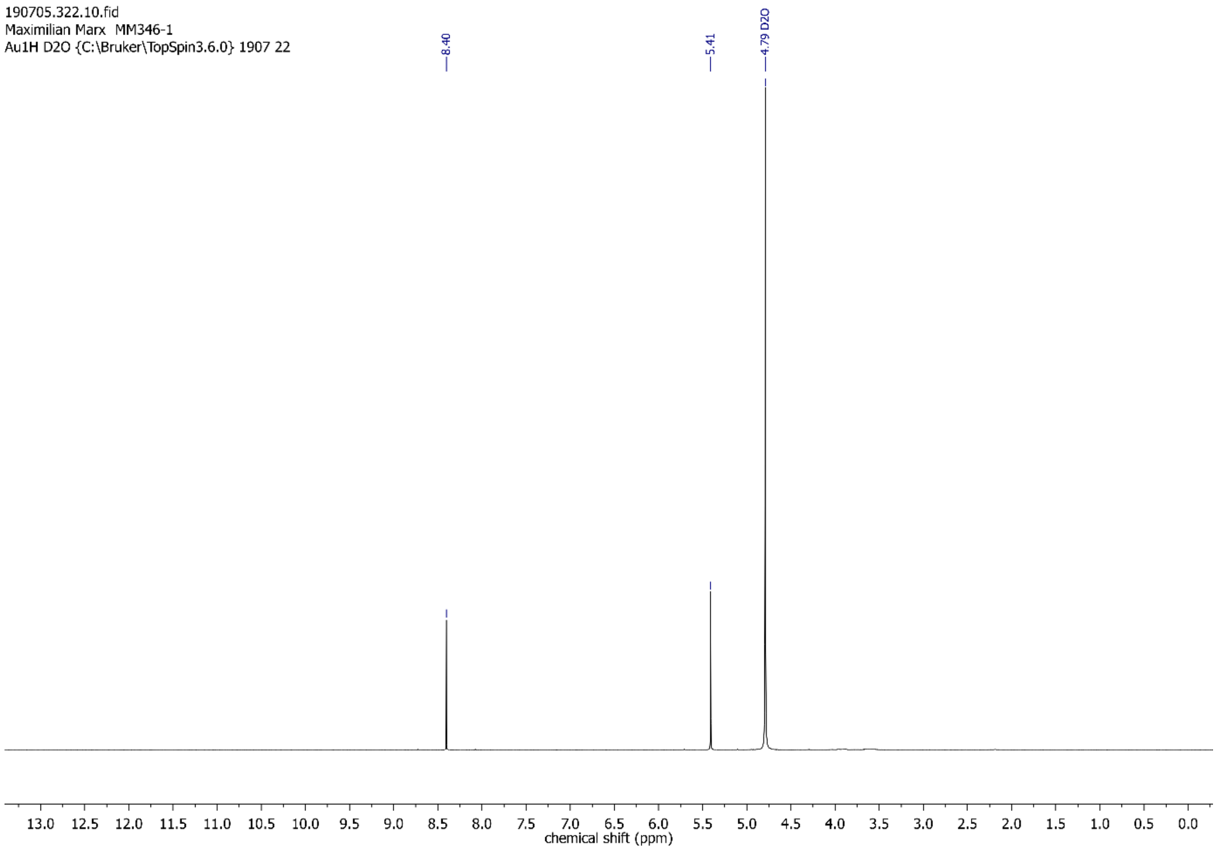

Supplementary Figure 15 | ^{1}H NMR spectrum (D_2O, 300 MHz) of the solid residue after NaOH mediated removal of the oxalate from complex 4 prepared by reaction of *in situ* formed complex 3 with O_2.

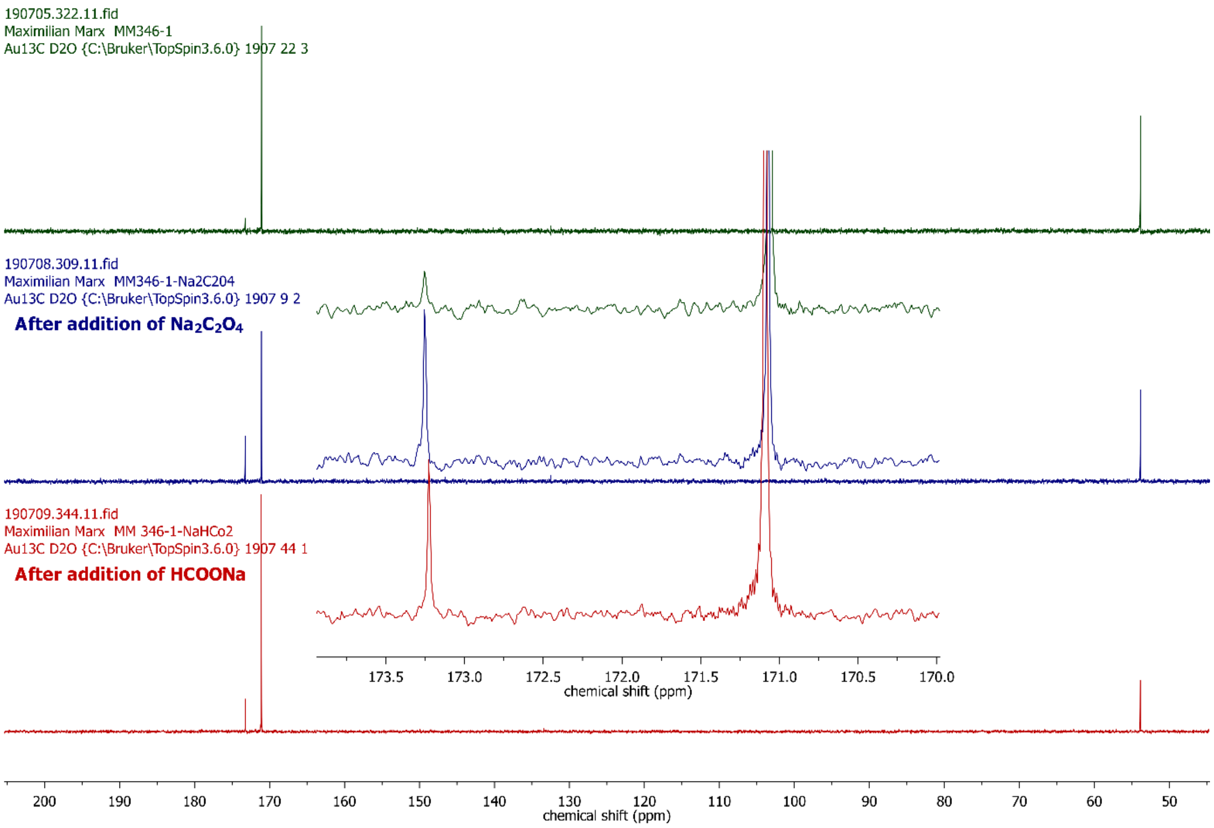

Supplementary Figure 16 | $^{13}C\{^1H\}$ NMR spectrum (D_2O, 75 MHz) of the solid residue after NaOH mediated removal of the oxalate from complex 4 prepared by reaction of *in situ* formed complex 3 with O_2.

Isolation of oxalate from complex 4 prepared by reaction of *in situ* generated 3 with air

The removal of oxalate was performed following the procedure described on p. S-19. To complex **4** (55.1 mg), prepared by reaction of *in situ* generated Cu(I) complex **3** starting from complex **1** and sodium ascorbate with air and subsequent slow evaporation of the DMF, was added toluene (2.0 mL) and 1 M aqueous NaOH solution (1.0 mL, 1.00 mmol). The reaction mixture was stirred at room temperature for 17 h, yielding a greenish suspension. Volatiles were evaporated under reduced pressure and the blue/green solid residue extracted with CH_2Cl_2 (2.0 mL). D_2O (0.7 mL) was added to the blue/green solid and the resulting suspension analysed by NMR spectroscopy (Supplementary Figure 17 and Supplementary Figure 18). To the same sample were added (after

complete NMR spectroscopic analysis) $Na_2C_2O_4$ and sodium formate as internal standards for direct determination of the oxalate/formate ^{13}C resonances.

^{1}H NMR (D_2O, 300 MHz): δ [ppm] = 8.40 (s, HCO_2Na), 5.41 (residual CH_2Cl_2).

$^{13}C\{^1H\}$ NMR (D_2O, 75 MHz): δ [ppm] = 173.2 ($Na_2C_2O_4$), 171.1 (HCO_2Na).

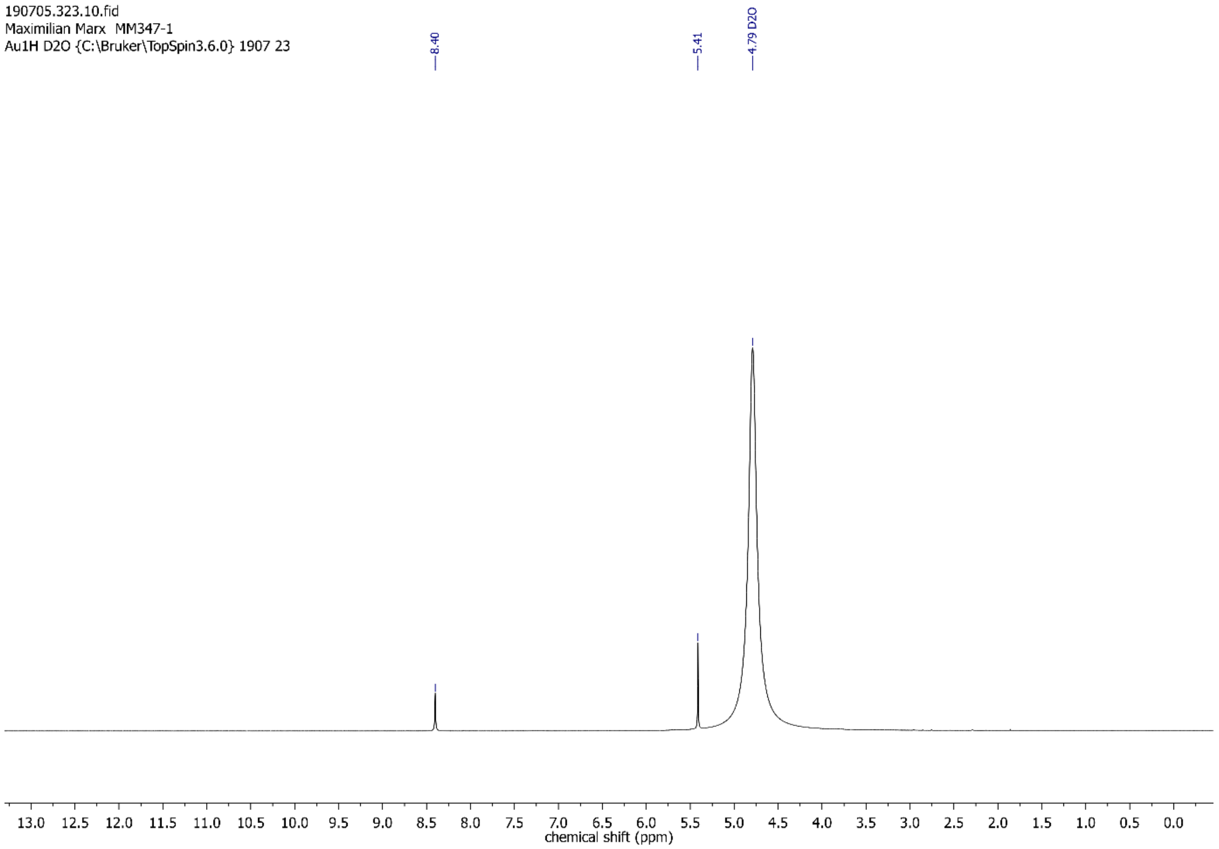

Supplementary Figure 17 | ^{1}H NMR spectrum (D_2O, 300 MHz) of the solid residue after NaOH mediated removal of the oxalate from complex 4 prepared by reaction of *in situ* formed complex 3 with air.

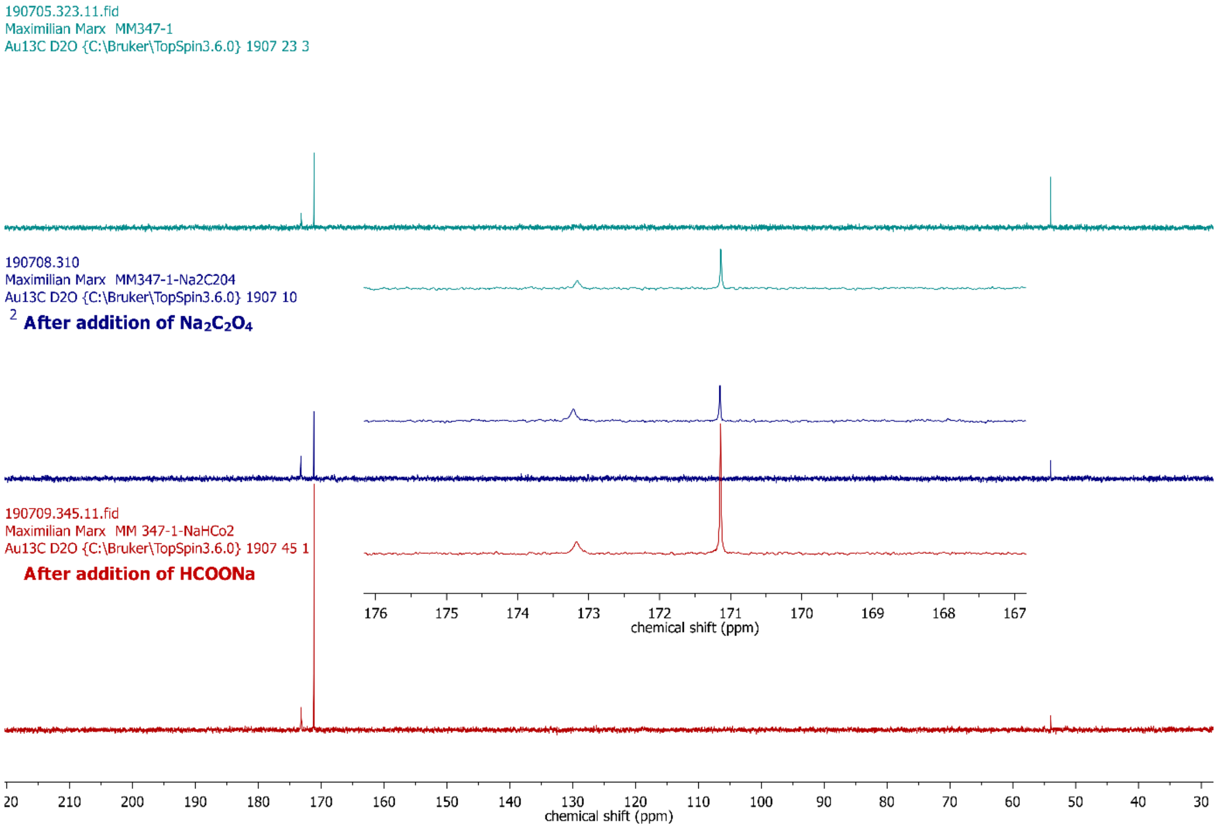

Supplementary Figure 18 | $^{13}C\{^1H\}$ NMR spectrum (D_2O, 75 MHz) of the solid residue after NaOH mediated removal of the oxalate from complex 4 prepared by reaction of *in situ* formed complex 3 with air.

Isolation of oxalate from complex 4a prepared from 1 and $(Bu_4N)_2C_2O_4$

The oxalate complex **4a** was prepared by reaction of **1** with $(Bu_4N)_2C_2O_4$ as described in the previous publication[4]. It was treated with NaOH to remove oxalate, following a literature procedure[7].

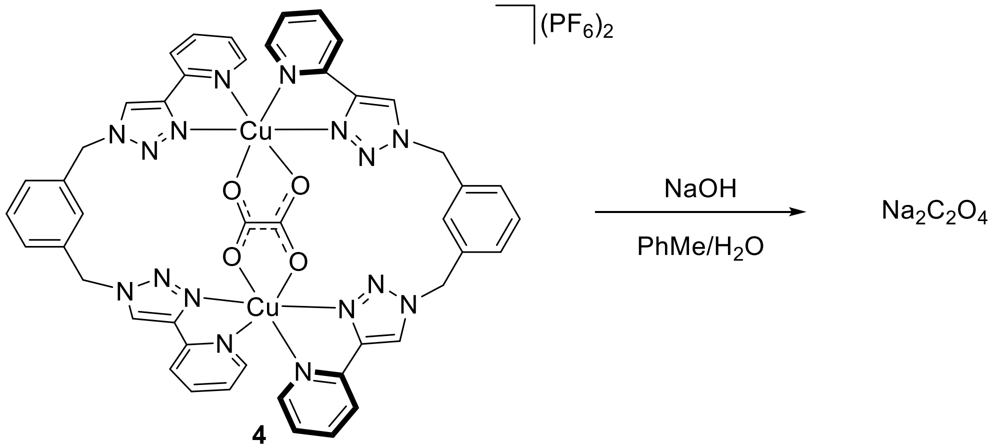

4

4 (29.3 mg, 22.6 µmol, 1.00 equiv.) was suspended in toluene (1.0 mL) and 1 M aqueous NaOH solution (0.50 mL, 0.50 mmol, 22 equiv.) was added. The suspension was stirred at room temperature for 16 h, giving a bluish suspension. Volatiles were evaporated under reduced pressure at 50 °C and the resulting brown/black residue extracted with CH_2Cl_2 (1.0 mL). The solid residue was dissolved/suspended in D_2O (0.6 mL) and analyzed by NMR spectroscopy (Supplementary Figure 19 and Supplementary Figure 20). The ^1H spectrum indicates that no proton-containing products were isolated by this procedure.

^{1}H NMR (D_2O, 400 MHz): δ [ppm] = 5.44 (residual CH_2Cl_2)

^{13}C{^{1}H} NMR (D_2O, 101 MHz): δ [ppm] = 53.9 (residual CH_2Cl_2), 173.3 ($Na_2C_2O_4$; identical to the signal from commercial $Na_2C_2O_4$ added to the sample in the second spectrum).

Identical results were obtained when this procedure was applied to samples of **4** prepared from *in situ* generated **3** by reaction with air or O_2. For details, see p. S-21.

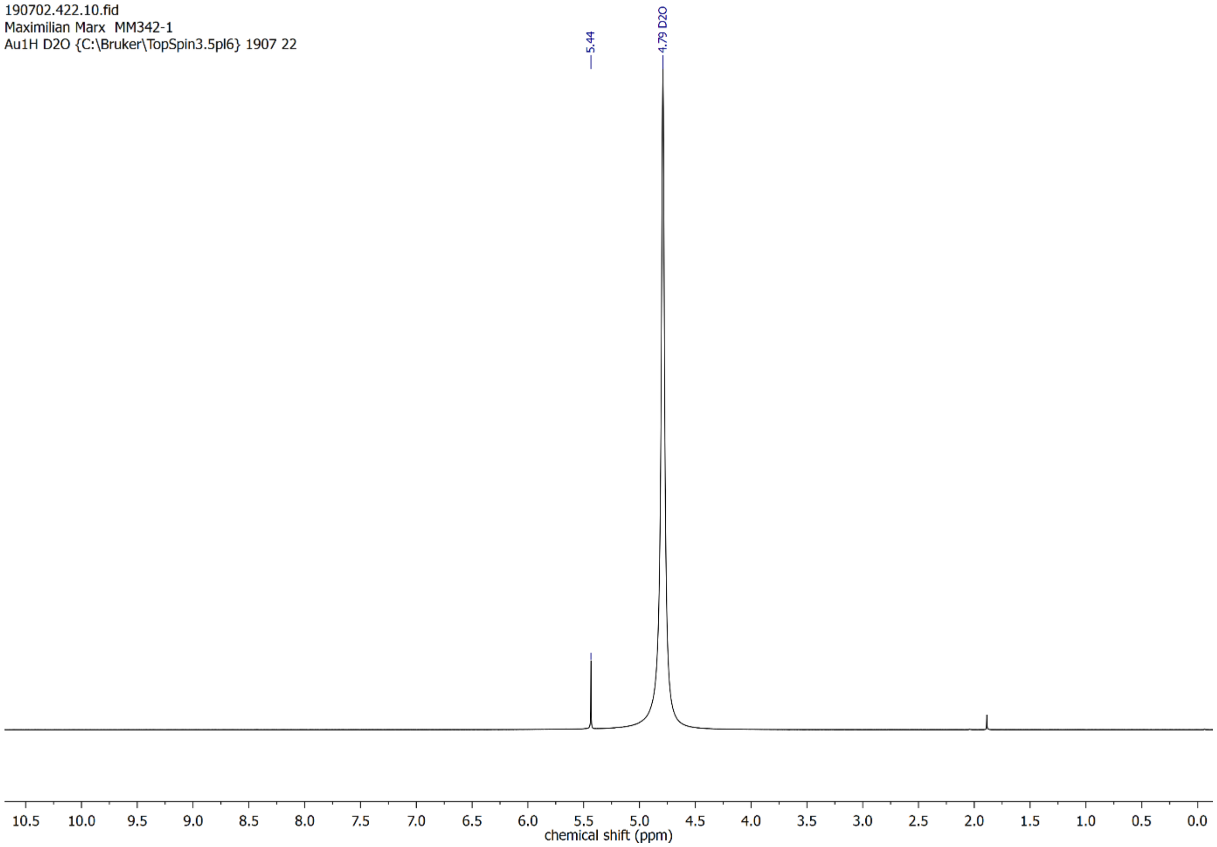

Supplementary Figure 19 | ^{1}H NMR spectrum (D_2O, 400 MHz) of the solid residue after NaOH mediated removal of the oxalate from complex 4 prepared by reaction of complex 1 with $(Bu_4N)_2C_2O_4$.

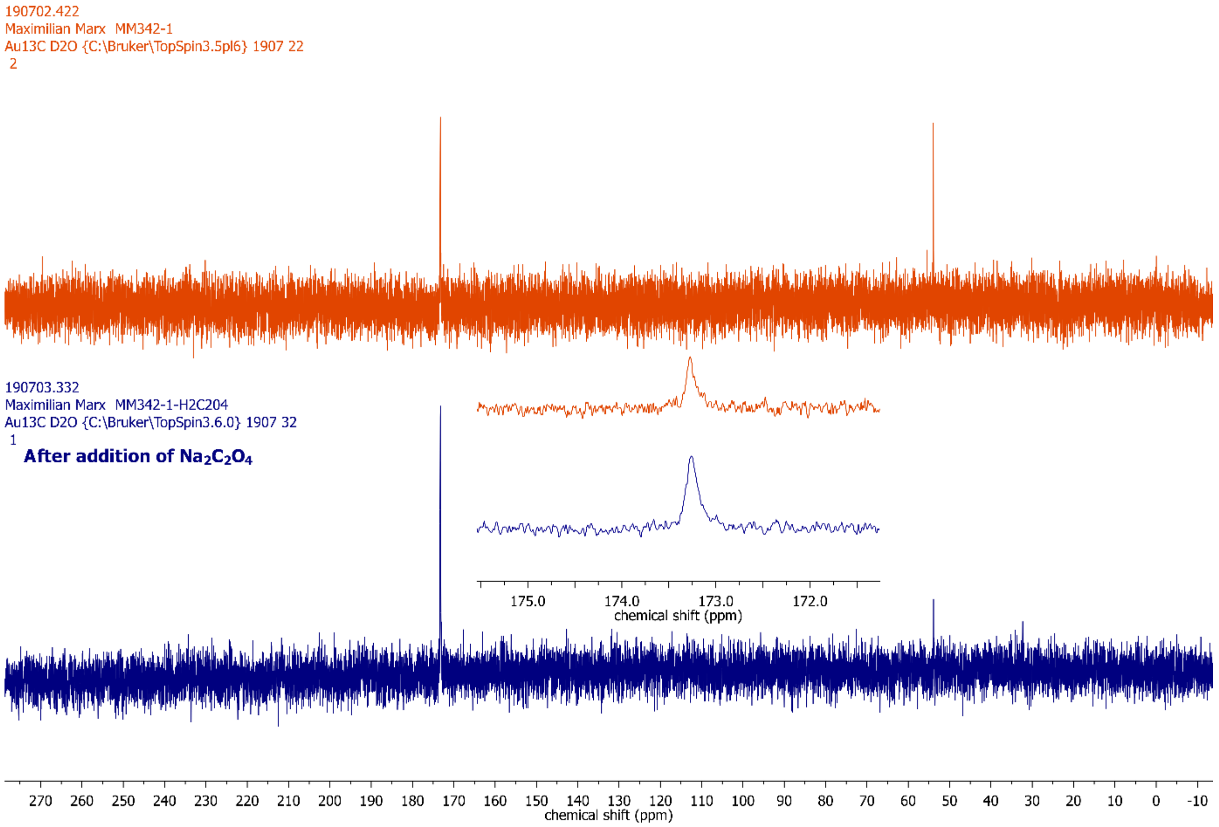

Supplementary Figure 20 | $^{13}C\{^1H\}$ NMR spectrum (D_2O, 101 MHz) of the solid residue after NaOH mediated removal of the oxalate from complex 4 prepared by reaction of complex 1 with $(Bu_4N)_2C_2O_4$.

Quantum chemical (DFT) calculations

All calculations employed Gaussian 16, version A.03, running on Windows computers, with the B3LYP functional and a mixed basis set (6-31G(d) on metal atoms and 6-311G(d,p) on all other atoms), as used by de Almeida et al.[9] in their study of Cu(acac)$_2$. The species calculated were [Cu$_2$(*m*-xpt)$_2$(μ-C$_2$O$_4$)]$^{2+}$ (singlet and triplet; no symmetry assumed) and [Zn$_2$(*m*-xpt)$_2$(μ-C$_2$O$_4$)]$^{2+}$ (singlet; C_{2h} symmetry). The calculation of the triplet state of [Cu$_2$(*m*-xpt)$_2$(μ-C$_2$O$_4$)]$^{2+}$ did not converge; results obtained for the triplet with 6-31G(d) basis set on all atoms are included below. The starting coordinates for both species were those of our published structure of [Cu$_2$(*m*-xpt)$_2$(μ-C$_2$O$_4$)](PF$_6$)$_2$·4CH$_3$CN (**4a**). Final M···M distances were 5.245 Å (Cu, singlet), 5.210 Å (Cu, triplet), and 5.424 Å (Zn), compared to 5.4213(7), 5.462(2), and 5.2123(6) Å in the X-ray analyses of **4**, **4a**, and **4b**, respectively. Vibrational frequency calculations performed with the optimized coordinates gave all positive real frequencies. Frequencies calculated for the asymmetric (IR-active) CO stretching vibration are presented in Supplementary Table 2.

Supplementary Table 2 | IR frequencies $\tilde{\nu}_{asym}$(CO) based on DFT electronic structure calculations

M	Spin	[M$_2$(*m*-xpt)$_2$(μ-C$_2$O$_4$)]$^{2+}$	[M$_2$(*m*-xpt)$_2$(μ-(^{13}C$_2$O$_4$))]$^{2+}$	Shift, $\Delta\tilde{\nu}$
Cu	singlet	1624.1 cm^{-1}	1579.0 cm^{-1}	−44.9 cm^{-1}
Cu	triplet[a]	1728.6 cm^{-1}	1681.8 cm^{-1}	−46.8 cm^{-1}
Zn	singlet	1703.9 cm^{-1}	1658.2 cm^{-1}	−45.7 cm^{-1}

[a]Smaller basis set used in calculation; see text for details.

The new IR and MS experiments show the spectral properties of genuine labeled **4**, and demonstrate conclusively that it does not form when ^{13}CO$_2$ is used. In the IR spectrum (Supplementary Figure 8), the band at ca. 1668 cm^{-1} is due to DMF in the crystal; it is the band at 1639 cm^{-1} that clearly shifts to lower energy on ^{13}C substitution. This shift of −39 cm^{-1} is close to the values we estimate here *via* DFT calculations (ca. −45 cm^{-1}; see Supplementary Table 2).

These values are also close to those for polymeric $[(bpy)Cu(\mu\text{-}C_2O_4)]_\infty$ (observed shift, −30 cm^{-1}; calculated, −37 cm^{-1})[10] and for aqueous $Na_2C_2O_4$ (observed shift, −43 cm^{-1})[11].

X-ray crystallography

Intensity data were collected at low temperature on a Bruker Kappa Apex-II DUO CCD diffractometer fitted with an Oxford Cryostream chiller. Data reduction included absorption corrections by the multiscan method, with SADABS[12,13]. The structures were determined by direct methods and difference Fourier techniques and refined by full-matrix least squares, using SHELXL[14,15]. All non-hydrogen atoms were refined anisotropically. In the final model for **6**, one BF_4 anion is disordered over two orientations, and one DMF solvent molecule is disordered across a twofold axis. All H atoms were placed in idealized positions. For additional details, see Supplementary Table 3.

The IUCr CheckCIF report for the structure of **6** contained no A- or B-level alerts. The report for **4b** contained one B-level alert, that the value of $\sin(\theta_{max})/\lambda$ (0.568) is less than the minimum recommended value of 0.575. This very small difference is attributable to difficulty in accessing data near the maximum θ limit with Cu radiation on a dual-wavelength diffractometer. This explanation is included in the uploaded CIF file via a Validation Reply Form.

In addition to the new structures described in this work, X-ray crystallography was used to identify **4** as a product in the following reactions: (i) **3** + DHA *in situ* + air; (ii) **3** + DHA *in situ* + O_2 (no CO_2); and (iii) **1** + added DHA + air.

Supplementary Table 3 | Crystal data and structure refinement parameters for 6 and 4b

Compound	**6**	**4b**
CCDC deposition num	1976240	1976241
formula	$[Cu_3(m\text{-xpt})_3(\mu_3\text{-}CO_3)](BF_4)_4\cdot 2.5DMF$ ($C_{74.5}H_{71.5}B_4Cu_3F_{16}N_{26.5}O_{5.5}$)	$[Cu_2(m\text{-xpt})_2(\mu\text{-}C_2O_4)](BF_4)_2\cdot 2DMF$ ($C_{52}H_{50}B_2Cu_2F_8N_{18}O_6$)
M	1963.94	1323.80
crystal system	Monoclinic	Monoclinic
space group	*I*2/*a*	$P2_1/c$
a/Å	29.1381(9)	10.3437(4)
b/Å	17.6927(5)	24.0279(10)
c/Å	33.1192(14)	12.7325(5)
β/deg	99.0670(10)	111.179(3)
V/Å^3	16860.6(10)	2950.8(2)
Z	8	2
T/K	100.0(5)	90.0(5)
D_{calc}/g cm^{-3}	1.547	1.490
crystal dimensions/mm	0.51 x 0.36 x 0.22	0.10 x 0.040 x 0.020
Radiation	MoKα (TRIUMPH curved graphite monochromator)	CuKα (IμS microfocus; QUAZAR multilayer optics)
θ limits/deg	1.732 – 34.459	3.679 – 61.213

reflns, measd / unique / obsd	198022 / 35460 / 23758	17493 / 4481 / 3241
Data / params	35460 / 1213	4481 / 399
F(000)	7992	1352
μ/mm^{-1}	0.854	1.671
R_{int}	0.0524	0.0677
$R[I>2\sigma(I)]$	0.0626	0.0419
R_w (all data)	0.1767	0.1046
GOF	1.038	1.012

Common ascorbate oxidation products

Many studies of oxidation of ascorbic acid and ascorbate have been reported, as outlined in the main text of this article. The first isolable product is almost always dehydro-L-ascorbic acid (DHA). One of the major products of further oxidation of DHA is oxalate; L-threonate frequently accompanies it[16-19]. Oxidation steps involving these species are shown in Supplementary Figure 21.

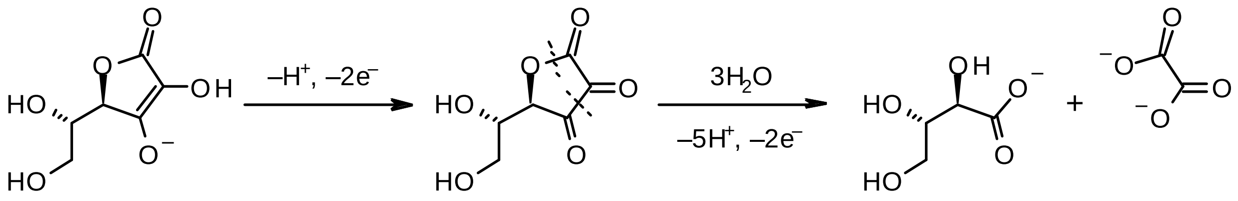

L-Ascorbate Dehydroascorbic acid L-Threonate Oxalate

Supplementary Figure 21 | Reactions illustrating the most commonly observed oxidation of ascorbate, and one of the more commonly observed further oxidations reported for dehydroascorbic acid (DHA).

Supplementary References

1 Yang, C.-T., Fu, Y., Huang, Y.-B., Yi, J., Guo, Q.-X. & Liu, L. Room-Temperature Copper-Catalyzed Carbon–Nitrogen Coupling of Aryl Iodides and Bromides Promoted by Organic Ionic Bases. *Angew. Chem. Int. Ed.* **48**, 7398-7401 (2009).

2 Crowley, J. D. & Bandeen, P. H. A multicomponent CuAAC "click" approach to a library of hybrid polydentate 2-pyridyl-1,2,3-triazole ligands: new building blocks for the generation of metallosupramolecular architectures. *Dalton Trans.* **39**, 612-623 (2010).

3 Pokharel, U. R., Fronczek, F. R. & Maverick, A. W. Cyclic pyridyltriazole–Cu(II) dimers as supramolecular hosts. *Dalton Trans.* **42**, 14064-14067 (2013).

4 Pokharel, U. R., Fronczek, F. R. & Maverick, A. W. Reduction of carbon dioxide to oxalate by a binuclear copper complex. *Nat. Commun.* **5**, 5883 (2014).

5 Fulmer, G. R., Miller, A. J. M., Sherden, N. H., Gottlieb, H. E., Nudelman, A., Stoltz, B. M., Bercaw, J. E. & Goldberg, K. I. NMR Chemical Shifts of Trace Impurities: Common Laboratory Solvents, Organics, and Gases in Deuterated Solvents Relevant to the Organometallic Chemist. *Organometallics* **29**, 2176-2179 (2010).

6 Nelson, I. V., Larson, R. C. & Iwamoto, R. T. Polarographic evidence for the stability of copper(I) ion in some non-complexing nonaqueous solvents. *J. Inorg. Nucl. Chem.* **22**, 279-284 (1961).

7 Takisawa, H., Morishima, Y., Soma, S., Szilagyi, R. K. & Fujisawa, K. Conversion of Carbon Dioxide to Oxalate by α-Ketocarboxylatocopper(II) Complexes. *Inorg. Chem.* **53**, 8191-8193 (2014).

8 Gaussian 16 Rev. A.03 (Wallingford, CT, 2016).

9 de Almeida, K. J., Cesar, A., Rinkevicius, Z., Vahtras, O. & Agren, H. Modelling the visible absorption spectra of copper(II) acetylacetonate by Density Functional Theory. *Chem. Phys. Lett.* **492**, 14-18 (2010).

10 Csonka, R., Kaizer, J., Giorgi, M., Reglier, M., Hajba, L., Mink, J. & Speier, G. Oxidative C-H and C-C bond cleavage by a (2,2′-bipyridine)copper(I) chloride complex. *Inorg. Chem.* **47**, 6121-6123 (2008).

11 Kuroda, D. G. & Hochstrasser, R. M. Two-dimensional infrared spectral signature and hydration of the oxalate dianion. *J. Chem. Phys.* **135**, 204502 (2011).

12 Krause, L., Herbst-Irmer, R., Sheldrick, G. M. & Stalke, D. Comparison of silver and molybdenum microfocus X-ray sources for single-crystal structure determination. *J. Appl. Crystallogr.* **48**, 3-10 (2015).

13 SADABS-2014 (University of Göttingen, 2014).

14 Sheldrick, G. Crystal structure refinement with SHELXL. *Acta Cryst. C* **71**, 3-8 (2015).

15 SHELXL-2014 (University of Göttingen, 2014).

16 Herbert, R. W., Hirst, E. L., Percival, E. G. V., Reynolds, R. J. W. & Smith, F. 299. The constitution of ascorbic acid. *J. Chem. Soc.* 1270-1290 (1933).

17 Shin, D. B. & Feather, M. S. The Degradation of L-Ascorbic Acid in Neutral Solutions Containing Oxygen. *J. Carbohyd. Chem.* **9**, 461-469 (1990).

18 Kurata, T., Miyake, N. & Otsuka, Y. Formation of L-Threonolactone and Oxalic Acid in the Autoxidation Reaction of L-Ascorbic Acid. *Biosci. Biotech. Biochem.* **60**, 1212-1214 (1996).

19 Dewhirst, R. A. & Fry, S. C. The oxidation of dehydroascorbic acid and 2,3-diketogulonate by distinct reactive oxygen species. *Biochem. J.* **475**, 3451-3470 (2018).
